# Using prosocial behavior to safeguard mental health and foster emotional well-being during the COVID-19 pandemic: A registered report of a randomized trial

**DOI:** 10.1371/journal.pone.0272152

**Published:** 2022-07-28

**Authors:** Andrew Miles, Meena Andiappan, Laura Upenieks, Christos Orfanidis

**Affiliations:** 1 Department of Sociology, University of Toronto, Toronto, Canada; 2 Institute of Health Policy, Management, and Evaluation, University of Toronto, Toronto, Canada; 3 Department of Sociology, Baylor University, Waco, TX, United States of America; 4 Ontario Institute for Studies in Education, University of Toronto, Toronto, Canada; University of Helsinki, FINLAND

## Abstract

**Background:**

The COVID-19 pandemic, the accompanying lockdown measures, and their possible long-term effects have made mental health a pressing public health concern. Acts that focus on benefiting others—known as prosocial behaviors—offer one promising intervention that is both flexible and low cost. However, neither the range of emotional states prosocial acts impact nor the size of those effects is currently clear—both of which directly influence its attractiveness as a treatment option.

**Objective:**

To assess the effect of prosocial activity on emotional well-being (happiness, belief that one’s life is valuable) and mental health (anxiety, depression).

**Methods:**

1,234 respondents from the United States and Canada were recruited from Amazon’s Mechanical Turk and randomly assigned (by computer software) to perform prosocial (N = 411), self-focused (N = 423), or neutral (N = 400) behaviors three times a week for three weeks. A follow-up assessment was given two weeks after the intervention. Participants were blind to alternative conditions. Analyses were based on 1052 participants (N_prosocial_ = 347, N_self_ = 365, N_neutral_ = 340).

**Findings:**

Those in the prosocial condition did not differ on any outcome from those in the self-focused or neutral acts conditions during the intervention or at follow-up, nor did prosocial effects differ for those who had been negatively affected socially or economically by the pandemic (all *p*’s > 0.05). Exploratory analyses that more tightly controlled for study compliance found that prosocial acts reduced anxiety relative to neutral acts control (β = -0.12 [95% CI: -0.22 to -0.02]) and increased the belief that one’s life is valuable (β = 0.11 [95% CI: 0.03 to 0.19]). These effects persisted throughout the intervention and at follow-up.

**Conclusion:**

Prosocial acts may provide small, lasting benefits to emotional well-being and mental health. Future work should replicate these results using tighter, pre-registered controls on study compliance.

## Introduction

COVID-19 has elicited an unprecedented global response to safeguard the physical health of vulnerable populations around the world. In the early months of 2020, national and local governments implemented strict measures to minimize contact between individuals in an effort to curb the spread of infection. During this time, people were asked to limit their physical interactions with others, stay inside their homes, and reduce both professional and personal ties that could not be maintained at a distance. The success of this approach slowed the spread of infection and reduced hospitalizations, but the price of isolation on mental health was high. Prevalence rates of anxiety, depression, and psychological stress increased in the American population during the pandemic [1–3]. Patterns of worsening mental health have also been documented in Canada [4], the United Kingdom [5], and in Chinese [6] and Dutch contexts [7]. There has long been a need to safeguard the mental health and emotional well-being of individuals in precarious circumstances, but the COVID-19 pandemic and its social and economic aftereffects have made this need even more pressing. Trained mental health providers are in short supply and likely to remain so as societies begin to experience the long-term effects of the COVID-19 response. There is accordingly a strong need for efficient, effective, and low-cost strategies for preserving mental health that can be deployed rapidly and widely. One highly portable solution would be to develop self-guided and home-based interventions [8,9].

Acts that focus on benefiting others—known as prosocial behaviors—offer one promising approach. The benefits of prosocial activities have been well-supported in the literature. Prosocial acts have been shown to boost a number of mental states including life satisfaction, well-being, and psychological flourishing. These effects can last for several weeks or even months following the end of an intervention [10–15]. Evidence indicates that prosocial behaviors produce positive emotions and happiness even when performed at a distance, making them ideally suited to the current crisis [16–18]. Prosocial acts can be flexibly enacted in many circumstances and often at little to no cost, facts which give a prosocial intervention the potential to be implemented quickly and widely.

There are two major limitations to past work that must be addressed before prosocial activity can be recommended to address the mental health concerns associated with COVID-19. First, past research has focused overwhelmingly on positive affect and happiness, with less attention given to other positive states such as a sense of meaning in life, and to negative states including anxiety and depression. Second, current knowledge about the effectiveness of prosocial action is based predominately on small-sample studies that are likely underpowered to detect effects, and post-hoc efforts to correct for this fact suggest that the true effects of prosocial acts on emotions might be smaller than previously supposed [19,20]. It thus remains unclear 1) how extensive and 2) how large the effects of prosocial behavior are, both of which have direct implications for its attractiveness as a treatment option. This study will address both of these challenges by examining the effect of prosocial behavior on two indicators of emotional well-being (happiness and belief that one’s life if valuable) and mental health (anxiety and depression) during a three-week intervention in a large online sample with sufficient power to detect effects. Because lasting effects have considerable practical appeal, we focus in particular on whether effects persist both throughout the intervention period and at a five-week follow-up.

### Prosocial behavior’s effects on emotional well-being and mental health

Doing good feels good. In the last several decades, this simple maxim has been placed under scientific scrutiny and accumulated a sizable body of evidence that attests to its veracity. Two recent meta-analyses found that prosocial activities produce a small positive effect on “emotional well-being”—a catch-all term that includes happiness, eudaimonic well-being, positive affect, psychological flourishing, and the absence of negative emotions [17,21]. Prosocial effects have been observed among children as well as adults and in samples across the world [17,22–27]. Aknin et al. [22] speculate the “warm glow” of giving might be a universal component of human psychology.

The emotional benefits of giving suggest that prosocial acts can be used in interventions to improve mental health. However, the practical utility of prosocial behavior depends on its effectiveness relative to other possible activities. We propose that people who are suffering from low levels of positive emotion and/or high levels of negative emotion often default to doing nothing—that is, they continue to perform routine daily activities that are affectively neutral but neglect to engage in activities intended to improve their emotional well-being. Alternately, they try to improve their mood by engaging in personally enjoyable activities intended to gratify their own emotional needs. We accordingly suggest that the practical utility of prosocial behavior is directly tied to its ability to enhance emotional well-being and relieve mental distress relative to these comparison points.

Below, we outline theoretical expectations for the effects of prosocial behavior on happiness, sense of meaning, depression, and anxiety.

#### Happiness

Work to date indicates that prosocial behaviors produce greater emotional well-being relative to a neutral or no action control condition [13,28,29]. This may be because prosocial acts fulfill basic psychological needs for autonomy, competence, and relatedness [30,31], or possibly a need for morality [32]. Kind acts might also prompt positive thoughts and additional positive behaviors that further enhance well-being [11,33]. Although the mechanisms underlying prosocial effects on happiness are still being established, the basic relationship appears robust. We accordingly hypothesize that *prosocial acts will increase happiness relative to affectively neutral acts* (Hypothesis 1a).

Prosocial behavior may also have advantages over self-focused acts of personal gratification. Common wisdom suggests that self-gratification should generate positive emotions, and existing research bears this out [34–36]. However, studies of prosocial acts indicate that prosocial behaviors produce greater gains in positive emotion than self-focused actions. For example, those who give to others instead of themselves report higher rates of happiness, regardless of the amount of money or size of the gifts involved, and regardless of the source of the funds [37–40]. Similarly, those who perform kind acts for others enjoy greater emotional well-being than those engaging in self-focused acts [13]. The greater emotional benefits of prosocial purchases, in particular, might reflect the fact that money spent on experiences tend to produce larger and longer-lasting gains in happiness than material purchases [41,42]. Prosocial spending effects could accordingly arise because they produce positive experiences rather than the acquisition of material goods. Another possibility is that individuals performing personally enjoyable acts might see themselves as self-indulgent, leading to mixed emotions—enjoyment from the self-gratification, but also negative emotions like guilt from a perceived sense of selfishness [43,44]. Regardless of the underlying mechanisms, we suggest that *prosocial acts will increase happiness relative to self-focused acts intended to gratify personal emotional needs* (Hypothesis 1b).

#### Sense of meaning

Humans have a desire to perceive and preserve meaning in life [45,46]. Meaning in life can be divided into (at least) three aspects: coherence (life makes sense), purpose (direction and goals in life), and significance (life is valuable and worth living) [47,48]. While there are reasons to suppose that prosocial action might influence any of these aspects, we focus on significance because we suspect that evaluations of one’s life are more responsive to changing life circumstances—such as those brought on by the COVID-19 pandemic—than are the beliefs that allow individuals to make sense of life, or the long-term goals they hold. Additionally, far less research has examined significance, making the need to understand it more acute [47].

According to Baumeister [49], people find a sense of meaning when they believe that their actions are “right and good and justifiable” (p. 36). Meaning might also arise from the sense of belonging that accompanies positive social connections [47,50,51]. Prosocial acts could generate a sense of meaning through either mechanism: they are widely considered to be “right and good”, and they could initiate positive interactions that lead to lasting social connections.

Empirical evidence linking prosocial acts to meaning in life is limited, but consistent with these claims. For example, recent studies by Van Tongeren, Green, Davis, Hook and Hulsey [52] found that engaging in altruistically motivated prosocial behavior is associated with greater meaning in life. These studies used undergraduate student samples, but the same effects have been found in the few studies that have examined adult samples [53,54]. Although a close examination of these studies reveals that they predominately examined purpose in life rather than significance, the positive correlation between these two aspects suggests that significance will respond to prosocial actions in similar ways [55]. We therefore hypothesize that *prosocial acts will increase a sense that one’s life has value relative to affectively neutral acts* (Hypothesis 2a).

Self-focused acts are unlikely to offer the same benefit. Although these behaviors can produce positive emotions, we suspect that they will not typically be viewed as “right and good.” More often, they will be perceived as morally neutral or even morally suspect to the extent that they are seen as selfish. Self-focused behaviors are also unlikely to lead to interpersonal connections that foster a sense of meaning in life, nor to a sense of attachment to something greater than oneself, which would seem to require attending to concerns beyond the self. We therefore hypothesize that *prosocial acts will increase a sense that one’s life has value relative to self-focused acts intended to gratify personal emotional needs* (Hypothesis 2b).

#### Depression

There are several reasons to expect that prosocial behavior will reduce depressive symptoms. Raposa and colleagues [56] argue that engaging in prosocial behavior can offset the impact of daily life stress on negative affect, which is a hallmark of depression. Depressed individuals also frequently hold negative views of themselves, such as the belief that they are unworthy or ineffective [57]. Prosocial actions could alleviate such judgments by shifting attention away from the self and towards the needs of others. Acts of personal gratification are unlikely to have this effect because they direct focus toward the self. Moreover, research suggests that symptoms of depression include increased interpersonal sensitivity and fear of social disapproval, both of which might prompt difficulty in interacting with others [58,59]. Engaging in acts of kindness towards others may lessen such concerns. Indeed, some evidence suggests that prosocial behaviors promote social integration and bonding with others [60], which may in turn prompt reciprocal supportive acts that could alleviate fears and reduce depression [61]. Self-focused acts might not confer this benefit because they often occur in isolation and are unlikely to prompt positive reciprocating behavior from others.

A handful of studies provide initial evidence that prosocial acts reduce depressive symptoms. Two studies indicate that individual who regularly engage in kind acts feel better on days when they help strangers [56] or friends [62]. A large cross-national study further found that those who volunteered reported lower levels of depression than those who did not [63]. In two experimental studies, engaging in kind or compassionate acts decreased depression relative to those in an affectively neutral control condition. These benefits persisted from one month to six months following the end of the intervention [64,65]. Given existing evidence and the theoretical considerations offered above, we hypothesize that *prosocial acts will reduce depression relative to both affectively neutral acts* (Hypothesis 3a) *and self-focused acts intended to gratify personal emotional needs* (Hypothesis 3b).

#### Anxiety

Prosocial acts could also reduce anxiety. Taylor, Lyubomirsky, and Stein [66] noted that positive emotions can reduce the physiological and psychological impact of negative emotions and argued that positive emotions might therefore be an effective treatment for depression and anxiety. Using a sample of individuals suffering from depression or anxiety, they found that those who engaged in positive activities—including prosocial acts—experienced significant improvements in levels of anxiety and depression compared to a wait-list control group. Similar results have been found in other samples of anxious individuals. In these studies, individuals who performed kind acts saw improvements in anxiety and a reduction in social avoidance, an anxiety-related behavior [10,67,68]. In contrast, self-focused behavior has been associated with increases in anxiety over time [69]. This may be because self-focused acts can elicit negative emotional reactions such as anxiety, sadness, or guilt if individuals believe that they should not be focusing on themselves or using valuable resources to satisfy their own desires [13,44]. Existing evidence therefore suggests that prosocial acts can reduce anxiety and might be more effective at doing so than self-focused acts. We therefore hypothesize that *prosocial acts will reduce anxiety relative to both affectively neutral acts* (Hypothesis 4a) *self-focused acts intended to gratify personal emotional needs* (Hypothesis 4b).

The foregoing discussion suggests that there are both theoretical and empirical reasons to believe that prosocial behavior will enhance emotional well-being and improve mental health. However, in several key regards the evidence for prosocial effects is still quite thin. There are relatively few studies linking prosocial behavior to sense of meaning, depression, and anxiety, for instance, and what studies do exist often use undergraduate students or samples selected for depression or anxiety. It is therefore unclear how robust and generalizable these results are. Further, studies of prosocial effects likely overestimate effect sizes due to their reliance on relatively small sample sizes (e.g., N per condition < 100) [19,70]. When studies are sufficiently powered, effect sizes can be much smaller [43, see also 24]. Our study addresses this evidence gap by estimating the effects of kind acts on happiness, sense of meaning, depression, and anxiety in a sample that is large enough to detect even modest effects.

Establishing the range and effectiveness of prosocial acts is an important first step in determining their possible therapeutic applications. However, some work suggests that improving well-being through positive activities (including prosocial behavior) requires considerable intentionality and effort [71]. A person’s ability to muster this effort could depend on the extent to which they must deal with additional life challenges. Additionally, the loss or restructuring of social relationships brought on by COVID-19 countermeasures might lead individuals to perform acts that are less likely to build relationships, which in turn might diminish their emotional effects [72] [but see 18]. In either case, the implication is that those most affected by the COVID-19 pandemic might be least capable of benefiting from prosocial behavior. To test this, our study examines whether any observed effects differ depending on how severely individuals were affected by the pandemic.

## Materials and methods

Table 1 summarizes key design elements of the study. Refer to the relevant sections of the manuscript for additional details and justifications of study procedures. Procedures followed this study’s pre-registered protocol (available at https://doi.org/10.1371/journal.pone.0245865, see also trial NCT04517006 on ClinicalTrials.gov), with the few deviations clearly noted. All materials to reproduce this study’s results are available at https://osf.io/63yg9/.

**Table 1:** Design table.

Question	Hypothesis	Sampling plan	Analysis Plan	Interpretation given to different outcomes	
Does prosocial behavior increase happiness?	1a. Prosocial acts will increase happiness relative to affectively neutral acts. 1b. Prosocial acts will increase happiness relative to self-focused acts intended to gratify personal emotional needs.	**Sample**: Canadian and American respondents from Amazon’s Mechanical Turk. **Design:** between subjects; respondents randomly assigned to complete prosocial acts, self-focused acts, or track activities 3 days/week for 3 weeks **Measurement intervals**: Respondents report activities on the three study days each week, and report happiness, valued life (an aspect of sense of meaning), depression, and anxiety at baseline, at the end of weeks 1, 2, 3, and at follow-up (week 5). **Target Sample size**: 360 per condition x 3 conditions = 1080, based on a power analysis targeting 95% power (*α* = 0.05, 2-tailed tests) **Sampling strategy**: • sample in batches so sampling rate can be adjusted to hit target sample size – adjust based on observed attrition • prevent responses from suspicious IP addresses • replace responses that meet our data exclusion criteria	**Analysis**: four random intercept models of the form: *y*_*it*_ = *μ*_*t*_ + ***γ***_*zt*_ ***z***_*i*_ + *α*_*i*_ + *ε*_*it*_ where *y*_*it*_ is the outcome of interest (happiness, valued life, depression, or anxiety), and ***z***_*i*_ includes both *p* (prosocial acts condition) and *s* (self-focused acts condition). The effects of interest are: ***γ***_*pt*_: the effects of *p* at each time point *t* ***γ***_*st*_: the effects of *s* at each time point *t* We will test all effects using Wald tests.	Hypothesis 1a, 2a, 3a, and 4a confirmed if:	
				*for*: *happiness*, *valued life*	*for*: *depression*, *anxiety*
				*γ*_p1_ > 0 *γ*_p2_ > 0 *γ*_p3_ > 0 *γ*_p5_ > 0	*γ*_p1_ < 0 *γ*_p2_ < 0 *γ*_p3_ < 0 *γ*_p5_ < 0
Does prosocial behavior increase the sense of meaning in life?	2a. Prosocial acts will increase a sense of meaning relative to affectively neutral acts. 2b. Prosocial acts will increase a sense of meaning relative to self-focused acts intended to gratify personal emotional needs.			Hypothesis 1b, 2b, 3b, and 4b confirmed if:	
				*for*: *happiness*, *valued life*	*for*: *depression*, *anxiety*
				*γ*_p1_ > *γ*_s1_ *γ*_p2_ > *γ*_s2_ *γ*_p3_ > *γ*_s3_ *γ*_p5_ > *γ*_s5_	*γ*_p1_ < *γ*_s1_ *γ*_p2_ < *γ*_s2_ *γ*_p3_ < *γ*_s3_ *γ*_p5_ < *γ*_s5_
Does prosocial behavior reduce depression?	3a. Prosocial acts will reduce depression relative to affectively neutral acts. 3b. Prosocial acts will reduce depression relative to self-focused acts intended to gratify personal emotional needs.			We will count a hypothesis as confirmed if the associated parameter is statistically significant at *p* < 0.05. Note that hypotheses might be confirmed at some time points but not others.	
Does prosocial behavior reduce anxiety?	4a. Prosocial acts will reduce anxiety relative to affectively neutral acts. 4b. Prosocial acts will reduce anxiety relative to self-focused acts intended to gratify personal emotional needs.				

### Ethics information

This study has been approved by the [name of ethics body redacted to allow double-blind peer review]. Informed consent was obtained from all research participants prior to beginning the study. Participants were paid for each component of the study that they completed. The compensation rate for each component was calculated based on its anticipated completion time, with a target pay rate of $14 CAD per hour (Ontario’s minimum wage). Study components included a baseline survey (15 min), nine daily surveys (3 min/each), and four follow-up surveys. The follow-up surveys were expected to take 10 minutes, but we calculated pay rates based on 10 minutes (survey 1), 12.5 minutes (surveys 2 and 3), and 15 minutes (survey 4) to discourage attrition. In total, participants could earn $21 CAD ($16 USD) if they completed all components of the study.

### Design

We examined the effects of prosocial behavior using a 3-week experimental intervention, followed by a follow-up assessment at 5 weeks. Tracking respondents over a 5-week period provides insights into how durable prosocial effects are. This intervention was embedded in a larger survey intended to address multiple questions regarding prosocial behavior and the COVID-19 pandemic. We focus solely on those parts of the survey relevant to the current, pre-registered intervention below.

**Fig 1:**
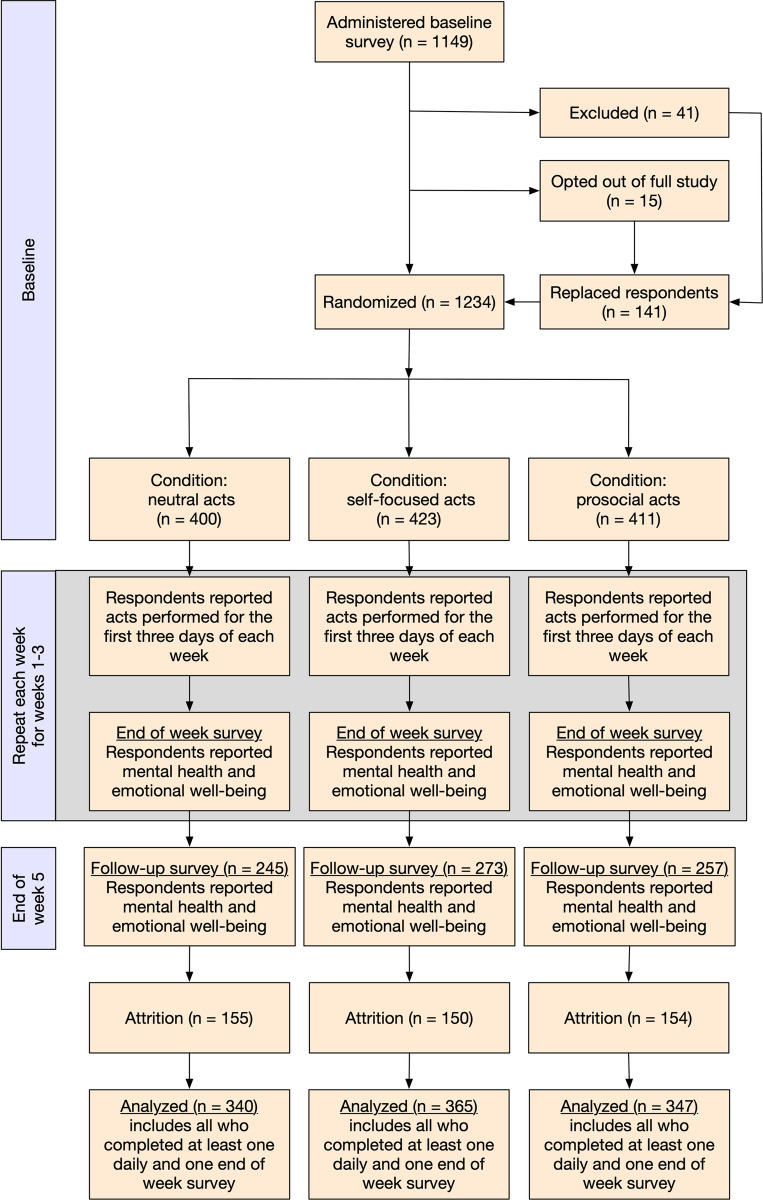
Flowchart of study procedures.

The study design is shown in Fig 1. At baseline we measured participants’ emotional well-being and mental health. Emotional well-being was assessed using happiness and feeling that one’s life is valuable, which is a facet of sense of meaning in life [55]. Mental health was measured as depression and anxiety. At the end of the baseline survey, we randomly assigned participants to one of three experimental conditions (using the randomization feature in Qualtrics survey software; between subjects design; respondents were blind to other conditions). In each condition, respondents were asked to perform certain types of behaviors for the first three days of each week. There are several reasons we chose to assign behaviors on the first three days each week. First, it represents a compromise between existing study designs that ask respondents to perform acts every day, and those that ask them to perform multiple acts on a single day. Prosocial effects have been found using both study designs. We also believed that a three-times-a-week approach would be less burdensome for respondents and therefore increase study compliance. Keeping the acts on consecutive days (i.e., the *first* three days of each week rather than any three days) should have made it easier for respondents to remember what to do and simplified survey administration.

One possible concern is that the effect of our intervention had worn off by the time we assessed our outcomes at the week’s end, particularly since our models control for baseline levels of these variables. However, assessing effects at a delay is consistent with our aim to determine whether prosocial acts can make lasting contributions to mental health and emotional well-being. Further, specifying that respondents perform acts on the *first* three days of each week makes it clear *when* respondents were performing prosocial acts, making it easier to judge whether effects really do last over the course of several days.

For our experimental conditions, one group of participants was asked to perform at least one prosocial act each day. This was our *prosocial intervention* condition. We compared these respondents to two control conditions. In our *neutral control* condition, we asked respondents to keep track of their daily activities, a task which we expected to be affectively neutral. Another group was instructed to perform a personally enjoyable act each day. We expected this *self-focused* condition to produce positive emotions, but consistent with past research we suspected that it would be less effective at doing so than our prosocial intervention. A full list of experimental prompts for each of our three experimental conditions can be found in S2 Appendix of the Supporting Information.

Respondents were drawn from Amazon’s Mechanical Turk and contacted using that platform’s built-in messaging capabilities for the first three days each week and asked to report what they did. At the end of weeks 1, 2, and 3 they completed a longer survey that repeated the same measures of emotional well-being and mental health used at baseline. At this point the intervention was complete. We recontacted respondent two weeks later (at the end of week 5) to assess whether the intervention had a lasting effect on mental health and emotional well-being. This final survey also included a measure of whether or not respondents continued their assigned behaviors following the end of the intervention. This measure might be useful for explaining any lasting effects in exploratory analyses.

### Measures

#### Experimental condition

Experimental conditions were coded using indicator variables for the prosocial intervention and self-focused conditions, with the neutral control as the reference category.

We used two commonly used indicators of *mental health*: depression and anxiety.

#### Depression

Depression was measured using the well-established 8-item short-form of the Centre for Epidemiological Studies-Depression Scale (CES-D) [73]. We asked respondents to report how often in the past week they (1) felt depressed, (2) felt that everything was an effort, (3) felt that sleep was restless, (4) felt happy (reverse coded), (5) enjoyed life (reverse coded), (6) felt lonely, (7) felt sad, and (8) could not get going. Responses were scored where 0 = “rarely or none of the time,” 1 = “some of the time,” 2 = “a moderate amount of time,” and 3 = “most or all of the time.” All items were summed to produce a final score. Consistent with past work, this scale had high alpha reliability scores: *α*_baseline_ = 0.91; *α*_week1_ = 0.92; *α*_week2_ = 0.91; *α*_week3_ = 0.91; *α*_week5_ = 0.91 [74,75].

#### Anxiety

We used the Hospital Anxiety and Depression Scale—Anxiety (HADS-A) scale to measure respondent anxiety, which is both commonly used and well-validated [76]. This is a 7-item scale that asks respondents how often in the past week they: (1) felt tense or wound up, (2) got a frightened feeling as if something awful was about to happen, (3) had worrying thoughts go through their mind, (4) got a frightened feeling like butterflies in the stomach, (5) felt restless as if they had to be on the move, (6) had a sudden feeling of panic, and (7) could sit at ease and feel relaxed (reverse coded). To ensure consistency with our measure of depression, responses were coded where 0 = “rarely or none of the time,” 1 = “some of the time,” 2 = “a moderate amount of time,” and 3 = “most or all of the time.” All items were summed to produce a final score (*α*_baseline_ = 0.87; *α*_week1_ = 0.88; *α*_week2_ = 0.86; *α*_week3_ = 0.89; *α*_week5_ = 0.88).

Both depression and anxiety were coded so that higher scores indicate greater levels of mental distress.

We also used two indicators of broader *emotional well-being*: subjective happiness and the sense that one’s life is valuable, which is a subset of the broader concept of a sense of meaning in life [55].

#### Subjective happiness

We used the well-validated Subjective Happiness Scale [77]. The items are: (1) “In general, I consider myself _______.” Responses options run from 1 = “not a very happy person” to 7 = “a very happy person.” (2) “Compared to most of my peers, I consider myself ________” with response options from 1 = “less happy” to 7 = “more happy.” (3) “Some people are generally very happy. They enjoy life regardless of what is going on, getting the most out of everything. To what extent does this characterization describe you?” (1 = “not at all” to 7 = “a great deal”). (4) “Some people are generally not very happy. Although they are not depressed, they never seem as happy as they might be. To what extent does this characterization describe you?” (1 = “not at all” to 7 = “a great deal”; reverse coded) These four items were averaged into a scale ranging from 1–7, where higher scores indicate greater subjective happiness (*α*_baseline_ = 0.92; *α*_week1_ = 0.92; *α*_week2_ = 0.93; *α*_week3_ = 0.94; *α*_week5_ = 0.94).

#### Valued life

We measured individual perceptions of whether one’s life has significance and value using the valued life subscale developed by Morgan and Farsides [55]. This measure consisted of the average of the following four items: (1) “My life is worthwhile,” (2) “My life is significant,” (3) I really value my life,” and (4) I hold my own life in high regard.” In each instance, response options ran from -3 = “strongly disagree” to 3 = “strongly agree” (*α*_baseline_ = 0.94; *α*_week1_ = 0.94; *α*_week2_ = 0.94; *α*_week3_ = 0.95; *α*_week5_ = 0.96). Steger et al. [78] also offer a widely used meaning in life scale, but this scale only measures the coherence and purpose aspects of meaning in life and not the value a person places on his/her life.

Our measures of depression, anxiety, happiness, and valued life are designed to capture relatively stable experiences of mental health and well-being rather than short term fluctuations. This is consistent with our aim to determine whether prosocial acts can produce long-term changes in these constructs. All non-dichotomous variables were standardized prior to analysis, making it possible to directly compare the size of experimental effects across outcomes. (Note that in the study protocol we failed to mention that dichotomous variables—the experimental condition indicators—would not be standardized).

### Sampling plan

Respondents were recruited from Canada and the United States using Amazon’s Mechanical Turk (MTurk). A short description of the study was posted to the MTurk website. Potential respondents (registered MTurk workers) could click to read a longer study description (see S1 Appendix in the Supporting Information for recruitment materials). Those interested could then proceed to the study. The study was open to all adults (age 18 and above).

Research indicates that anywhere from 5% to 25% of responses collected using MTurk offer low quality data [79,80]. Many of these responses come from respondents using virtual private servers (VPS) to access surveys they are not qualified for. Data quality can be substantially improved by blocking VPS users from taking surveys using techniques such as geolocation and IP address screening [79,81]. Accordingly, we collected data using the MTurk interface offered by CloudResearch which offers several data-quality safeguards [82]. In particular, we blocked participants that came from suspicious geocode locations (locations known or strongly suspected to be fraudulent) or that came from duplicate IP addresses. We informed all potential respondents that we were blocking VPS users so that legitimate respondents who might otherwise use a VPS had the option of deactivating their VPS and accessing the survey. As added precautions, we also used CloudResearch to filter out respondents whose IP addresses did not originate in the United States or Canada, and to block participants who had previously failed CloudResearch’s quality checks.

We were interested in the effects of prosocial acts on happiness, a sense that one’s life has value (valued life), depression, and anxiety. Few studies have examined the effects of prosocial behavior on valued life, depression, or anxiety. We therefore based our power calculations on studies of prosocial acts and happiness (including positive emotions). We drew estimates of effect sizes and sample sizes from a recent meta-analysis of prosocial behavior and emotions [17]. These estimates included the effects of prosocial acts compared to both neutral and self-focused behaviors. We excluded from consideration any effects from studies that sampled children or that used psychological flourishing as the outcome (as psychological flourishing is a broader concept than happiness). This left us with 37 effects.

Published studies of prosocial behavior likely suffer from small-sample bias [19]. To obtain a more accurate effect size for power calculations, we therefore used the “TOP10” heuristic proposed by Stanley et al. [83]. The TOP10 calculation is the average of the effect sizes in the top 10% of studies, where rankings are based on the reliability of the results. We used sample size as an indicator of study reliability. Applying TOP10 gave us 3.57 effects, which we rounded up to 4 to capture greater variety. These effects are: *d* = 0.08, 0.30, 0.20, and 0.18 [13,65,84]. The average of these four effects is *d* = 0.19. This is very similar to the estimate White et al. [19] give in their small-sample adjusted re-analysis of positive activity interventions (*r* = 0.10, which corresponds to *d* = 0.20).

We performed a power analysis to determine the necessary sample size to detect an effect size of *d* = 0.19 with 95% power (see supplemental files at https://osf.io/63yg9/ for more details). As described more fully below, our main analyses included controls for baseline levels of all outcome variables. We assumed that these variables would account for at least 50% of the variance in our outcomes, indicating a need for N = 357 or approximately 360 respondents per condition. The reasonableness of this assumption is indicated by large bivariate correlations between baseline and follow-up measures of emotional well-being and mental health in several studies. For example, Mongrain et al. [65] found that happiness measures correlated at 0.86 and depressive symptoms at 0.68 after one week. This indicates that the baseline measure of happiness alone would account for 0.86^2^ = 74% of the variance in happiness one week later, and that baseline depression would account for 0.68^2^ = 46% of the variance in depression one week later. Further, baseline happiness correlated with week 1 depression at -0.64, while baseline depression correlated with week 1 happiness at -0.63. This suggests that adjusting for baseline measures of *both* happiness and depression would almost certainly explain even more variation in week 1 measures. See studies such as Proyer et al. [85] and Manthey et al. [86] for similar correlations using different time frames. We expected a 30% attrition rate after baseline which means that we planned to sample 360/0.7 = 514 per condition [87].

We posted the baseline survey in two batches (January 2021). The first batch contained half the target sample (N = 771), and was used to gauge the attrition rate (see data exclusion criteria below). Attrition was low (3%), so we adjusted the sample size of the second batch down (to 350) to try and capture at least 350 respondents per experimental condition. To be clear, our stopping rule for recruiting sample participants did not require estimating any of the effects of interest in the study (i.e., it did not depend on the anticipated effect size). The only factor was whether we had obtained a sufficient number of respondents in each experimental condition.

We planned to begin sampling on a Sunday and repeat our sampling procedure each week if needed to obtain a sufficient sample. However, we obtained enough respondents the first day, so repeated sampling was not necessary. We did recruit a second sample (February 2021), but that was to replace respondents who had been excluded from the original sample, as described below.

#### Data exclusion criteria

Per our protocol, we excluded respondents from the study if any of the following applied:

1. A respondent completed the baseline study unrealistically quickly. We measured response speed using the average number seconds spent on each survey item (seconds per item, or SPI). Following Wood et al. [80], we judged a response to be unrealistically fast if its SPI value was less than 1. SPI calculations excluded optional items.
2. A respondent did not complete at least half of the items in the baseline survey.
3. A respondent provided off-topic, non-sensical (e.g., random or gibberish words), or non-English responses to an open-ended question in the baseline survey. The open-ended question followed a dictator game (that is not part of the pre-registered portion of the study) and asked respondents what they hoped to accomplish by acting as they did during the dictator game. The specific nature of this question made it straightforward to detect off-topic responses. A comment had to be judged as off-topic, non-sensical, or non-English by two members of the research team to be excluded.
4. A respondent did not agree to continue with the study when asked if they wish to continue taking part in the study at the end of the baseline study, or in private correspondence with the researchers.
5. A respondent completed the baseline study using an IP address from outside Canada or the United States, or that appeared to originate from a VPS or other suspicious source. The CloudResearch platform should have prevented these respondents from beginning the survey so we did not actively check location or VPS use. We included this exclusion criterion mainly as a precaution.
6. Technical difficulties prevented a respondent from completing the baseline study.

Because obtaining sufficient power to detect effects is a central aim of this study, we planned to replace respondents who were removed for reasons 1–5 in a rolling fashion. We excluded 56 respondents based on these criteria (see Fig 1). In the case of technical failure (#6), we planned to to resolve the issue and administer the baseline survey to the same participant if possible. However, this never occurred during the study.

#### Attrition during the study

We counted a respondent as having dropped out of the study if s/he failed to complete three surveys in a row (e.g., three daily surveys, two daily surveys and an end of week survey). We assessed attrition each week and did not send additional surveys to respondents who dropped out. We assessed attrition following each end of week survey, with one exception. Through an oversight we failed to examine attrition immediately following the first end of week survey, and instead assessed attrition several days into the 2^nd^ week. We retained several respondents who should have been eliminated following the end of week 1 survey but who began participating during the 2^nd^ week.

Respondents who dropped out were replaced unless they had completed at least one daily survey and one end-of-week survey. There were two reasons for this. First, this approach kept the costs of the study predictable. Second, with some data for both daily and end of week surveys the full-information maximum likelihood missing data handling we employed allowed us to recover some of the power lost through sample attrition. We originally planned to replace respondents each week but realized that it would be much simpler to simultaneously replace any respondent who had been excluded for any reason (i.e., based on the above exclusion criteria or through attrition). Accordingly, we drew a second sample about one month after recruiting original sampe (N = 179). Of this sample, 8 opted out of the full study and 30 were excluded, leaving 141 additional respondents to supplement the original sample (see Fig 1).

Baseline descriptive information for the final sample can be found in S6 Appendix.

### Statistical analysis plan

Our goal is to determine the effects of acting prosocially on happiness, valued life, depression, and anxiety. Each of these outcomes was measured at baseline, and at 1, 2, 3, and 5 weeks.

Past work has often assessed the effectiveness of prosocial interventions using t-tests or ANOVAs. However, these methods are inefficient when data exist that can explain a substantial amount of the variance in the outcome. Our data contain baseline measures of all outcomes, which we expected to be highly related to our outcomes at all time points, and consequently capable of increasing the efficiency of our estimates. We therefore included baseline measures of all outcome variables in each of our models.

We also assessed the level of compliance with experimental instructions. At the end of week three, respondents were asked to indicate how often they reported behaviors that they did not actually perform, and to indicate how much effort they put into performing acts that were beyond what they normally do (see S5 Appendix). After completing data collection but prior to conducting analyses, we examined whether compliance with experimental instructions varied across experimental conditions using chi-squared tests. Respondents did not vary across conditions with regard to how often they reported behaviors that they did not actually perform ($\chi_{8}^{2}$ = 9.55, *p* = 0.298), but they did vary with respect to how much effort they put into their tasks ($\chi_{8}^{2}$ = 366.02, *p* < 0.001). At first glance this is unsurprising—after all, respondents in the neutral acts control condition were instructed to not alter their routine and simply report their behavior. However, the pattern persisted when just considering those assigned to the prosocial and self-focused acts conditions ($\chi_{4}^{2}$ = 36.47, *p* < 0.001), with effort generally lower among those in the prosocial condition. Among these respondents, 54.5% reported that they never or occasionally tried to do something beyond what they normally do, compared to 32.2% in the self-focused condition. Per our pre-registration, we therefore included the amount of effort as a continuous control in analyses. However, we realized that the topmost category—“other”—was not appropriately captured by this control strategy, so we added a binary control to the model marking those few respondents who selected this option (N = 8). This was not part of the pre-registration but seemed a prudent precaution.

For each outcome, we estimated a random intercept model. Random intercept models adjust for the fact that each respondent contributes multiple data points to the analysis. These models are of the form:

$$y_{it}=\mu_{t}+\gamma_{zt}z_{i}+\alpha_{i}+\varepsilon_{it}$$

Here *y*_*it*_ is the outcome variable that varies over both individuals (*i*) and time points (*t*). In this case, *t* could take on values of 1, 2, 3, and 5 indicating data from weeks 1, 2, 3, and 5. *μ*_*t*_ is an intercept that is allowed to vary over time, and **γ**_*zt*_ are the coefficients of the variables ***z***_*i*_ that are allowed to differ at each time point. The ***z***_*i*_ themselves are time-constant variables and so differ only between individuals. In this study ***z***_*i*_ included indicators for which experimental condition respondents were assigned to as well as controls for baseline measures of all mental health and emotional well-being variables. *α*_*i*_ allows the intercept for each individual to vary, and *ε*_*it*_ is a residual term that varies over both individuals and time.

**Fig 2:**
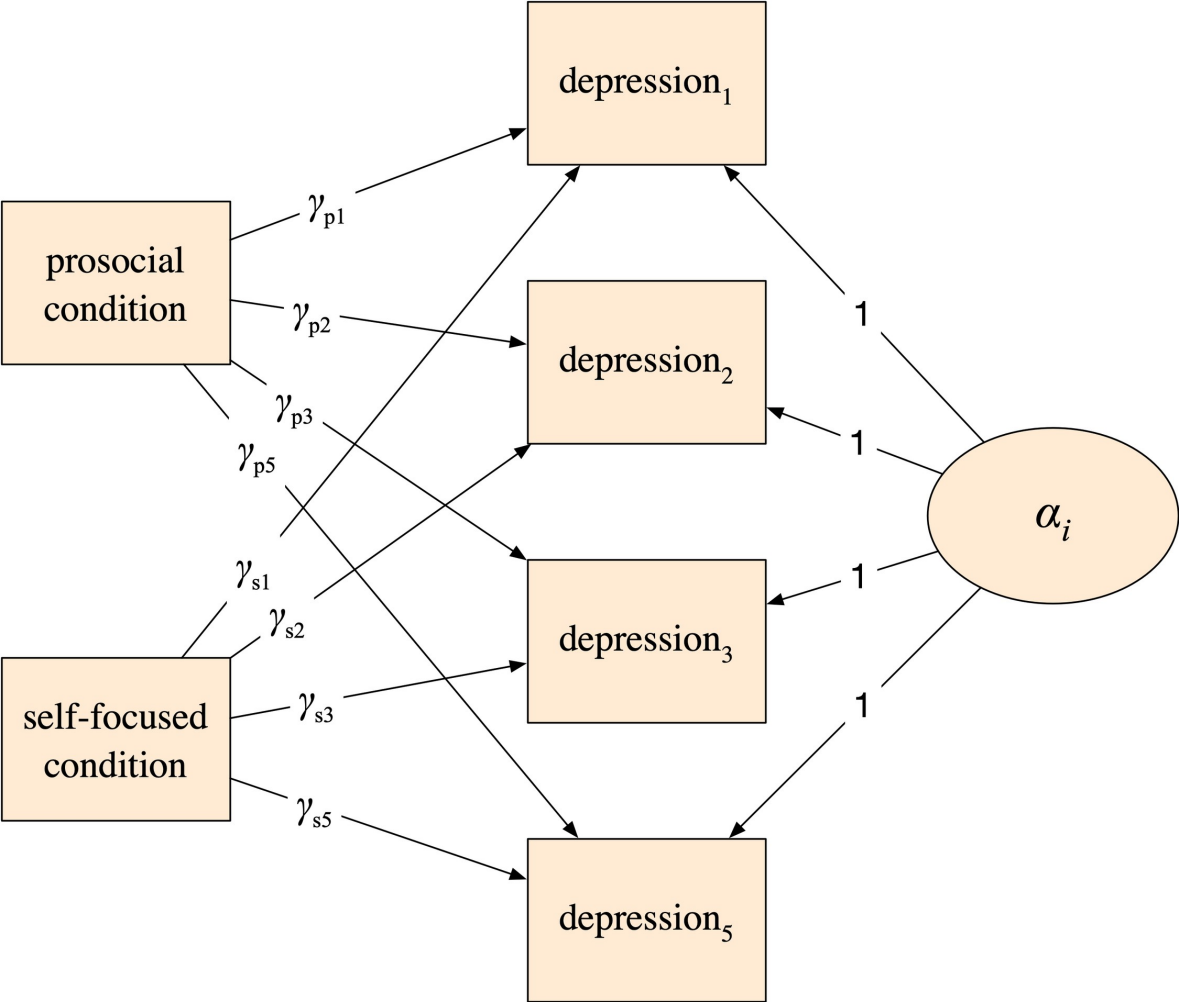
Example of a random intercept model as a structural equation model. Control variables, intercepts, and errors not shown for clarity.

We estimated all random intercept models using structural equation modeling (SEM) software [88]. This allowed us to use full-information maximum likelihood (FIML) to adjust for missing data in all analyses [89]. As an example, Fig 2 displays the random intercept model for depression shown in SEM form (controls, intercepts, and errors not shown). The γ_*p*_*’*s are the effects of the prosocial intervention, and the γ_*s*_*’*s are the effect of the self-focused condition. This model is maximally flexible, estimating a unique effect of each condition at each time point. This is roughly equivalent to estimating a t-test at each time point, but with the added power afforded by our control variables. Analogous models can be drawn for the remaining outcome variables. Our study hypotheses were evaluated by formally testing and comparing the coefficients from these models.

Table 2 lists the hypotheses we tested for each outcome, and how those hypotheses map onto the model. To interpret the hypothesized direction of effects, recall that a beneficial effect means *greater* emotional well-being and *fewer* mental health challenges. We tested all hypotheses using Wald tests (i.e., the tests on individual coefficients, or post-estimation linear hypothesis tests).

**Table 2:** Hypotheses for testing intervention effects.

Hypothesis	Confirmed if…	
	*for valued life*, *happiness*	*for depression*, *anxiety*
prosocial acts > neutral acts	*γ*_p1_ > 0 *γ*_p2_ > 0 *γ*_p3_ > 0 *γ*_p5_ > 0	*γ*_p1_ < 0 *γ*_p2_ < 0 *γ*_p3_ < 0 *γ*_p5_ < 0
prosocial acts > self-focused acts	*γ*_p1_ > *γ*_s1_ *γ*_p2_ > *γ*_s2_ *γ*_p3_ > *γ*_s3_ *γ*_p5_ > *γ*_s5_	*γ*_p1_ < *γ*_s1_ *γ*_p2_ < *γ*_s2_ *γ*_p3_ < *γ*_s3_ *γ*_p5_ < *γ*_s5_

We counted a hypothesis as confirmed if the associated parameter was statistically significant at *p* < 0.05. Note that in this approach hypotheses might be confirmed at some time points but not others (e.g., prosocial actions might have an effect on depression at week 1 but not at weeks 2, 3, or 5). While we acknowledge the possibility that effects might differ over time, we did not build predictions about changes over time into our hypotheses. This is consistent with the existing literature that has found prosocial effects following interventions of one to six weeks, and some work showing that these effects appear quickly and remain relatively constant over time [13].

We planned to conduct all analyses in Stata version 16 but switched to using R (version 4.0.3) with RStudio (version 1.4.1103) prior to conducting any analyses to take advantage of the powerful R markdown language for creating replication files with mixed commentary and code. SEM models were estimated using the lavaan package (version 0.6–7).

## Results

Table 3 presents the results of our planned hypothesis tests. Effects were consistently of negligible magnitude and non-significant at the *p* < 0.05 level. Thus, we failed to confirm Hypotheses 1a-4a, and 1b-4b. Our pre-registered analyses give no evidence that prosocial acts reduce depression and anxiety, nor do they improve happiness and a sense that one’s life if valuable compared either to self-focused acts or a neutral acts control condition consisting of reporting routine behaviors.

**Table 3:** Effects of prosocial behavior condition on depression, anxiety, happiness, and valued life.

	Prosocial vs. Control					Prosocial vs. Self-Focused				
Week	Estimate	SE	p	95% CI		Estimate	SE	p	95% CI	
**Depression**										
1	0.030	0.045	0.513	(-0.059,	0.119)	0.040	0.041	0.331	(-0.040,	0.119)
2	0.071	0.047	0.131	(-0.021,	0.164)	0.051	0.042	0.225	(-0.032,	0.135)
3	0.017	0.046	0.705	(-0.073,	0.108)	0.023	0.042	0.587	(-0.059,	0.105)
5	-0.006	0.052	0.913	(-0.108,	0.097)	0.043	0.047	0.353	(-0.048,	0.134)
**Anxiety**										
1	0.014	0.052	0.780	(-0.087,	0.116)	0.005	0.046	0.912	(-0.086,	0.096)
2	-0.046	0.056	0.411	(-0.156,	0.064)	-0.043	0.050	0.386	(-0.142,	0.055)
3	-0.027	0.055	0.627	(-0.134,	0.081)	-0.027	0.050	0.591	(-0.124,	0.071)
5	0.002	0.060	0.980	(-0.116,	0.119)	-0.017	0.053	0.745	(-0.122,	0.087)
**Happiness**										
1	-0.037	0.037	0.322	(-0.110,	0.036)	-0.013	0.033	0.686	(-0.079,	0.052)
2	-0.008	0.038	0.829	(-0.083,	0.066)	0.022	0.034	0.525	(-0.045,	0.088)
3	-0.052	0.036	0.145	(-0.122,	0.018)	-0.015	0.032	0.636	(-0.079,	0.048)
5	-0.017	0.040	0.666	(-0.095,	0.060)	0.018	0.035	0.602	(-0.051,	0.088)
**Valued life**										
1	-0.045	0.043	0.302	(-0.130,	0.040)	0.040	0.039	0.303	(-0.036,	0.116)
2	0.024	0.044	0.589	(-0.062,	0.109)	0.018	0.039	0.645	(-0.059,	0.095)
3	0.004	0.040	0.928	(-0.075,	0.082)	0.067	0.036	0.065	(-0.004,	0.138)
5	0.034	0.046	0.467	(-0.057,	0.125)	0.036	0.042	0.385	(-0.045,	0.117)

Note: Estimates are coefficients from random intercept models fit to each outcome and are allowed to vary across weeks of the study. Models controlled for baseline measures of all outcomes as well as the effort respondents reported putting into performing behaviors. The prosocial acts condition was compared to both the neutral acts (control) and self-focused acts conditions, and effects are standardized mean differences. N = 1052. All coefficient tests are two-tailed. Effects with *p* < 0.05 are bolded.

### Planned exploratory analyses

Per the protocol, we also assessed whether results differ for those who are more or less affected by the COVID-19 pandemic. We first investigated how much respondents reported (at baseline) that the pandemic had impacted their family life, non-family social life, employment, leisure time, and financial situation. These items formed a coherent scale (α = 0.63), though leisure time did not correlate as strongly with the other items and was therefore excluded (α = 0.65, after excluding leisure time). We then coded respondents who scored three or below on this scale as having been negatively affected by the pandemic (from “very negatively” to “somewhat negatively” affected on the original scale), those who scored five or above as positively affected (from “somewhat positively” to “very positively” affected on the original scale), and all others as minimally affected. Using this coding, 29.8% of the sample was negatively affected by the pandemic and 66.7% was minimally affected. Only 3.5% were positively affected, so we removed these respondents from analyses to avoid testing hypotheses with insufficient data.

We then refit our original analysis models but included interaction terms between experimental conditions and having been negatively affected by the pandemic (vs. minimally affected). Table 4 shows the marginal effects calculated from these models, along with the calculated differences between those who minimally and negatively affected by the pandemic. The only statistically significant effect is the difference term for the prosocial condition vs. the neutral acts control condition on a sense that one’s life if valuable, and then only at week 2. Given the number of tests performed and the lack of significant differences for valued life at other weeks, we suspect this single significant effect is likely due to chance and should not be interpreted. Thus, we have little evidence that performing prosocial acts has beneficial effects on mental health or emotional well-being, regardless of whether a person was or was not negatively affected by the COVID-19 pandemic.

**Table 4:** Marginal effects of prosocial behavior for groups minimally and negatively affected by the COVID-19 pandemic.

		Prosocial vs. Control					Prosocial vs. Self-Focused				
Week	COVID impact	Estimate	SE	p	95% CI		Estimate	SE	p	95% CI	
**Depression**											
1	minimal	0.044	0.054	0.418	(-0.062,	0.150)	0.016	0.049	0.751	(-0.081,	0.112)
1	negative	0.011	0.075	0.879	(-0.136,	0.159)	0.091	0.071	0.200	(-0.048,	0.231)
1	[neg.-min.]	-0.032	0.088	0.714	(-0.205,	0.141)	0.076	0.086	0.378	(-0.093,	0.244)
2	minimal	0.091	0.056	0.106	(-0.019,	0.202)	0.067	0.052	0.191	(-0.034,	0.169)
2	negative	0.047	0.080	0.555	(-0.109,	0.203)	0.022	0.076	0.773	(-0.127,	0.170)
2	[neg.-min.]	-0.044	0.093	0.636	(-0.227,	0.138)	-0.046	0.091	0.615	(-0.224,	0.132)
3	minimal	0.019	0.054	0.722	(-0.087,	0.126)	0.017	0.050	0.741	(-0.082,	0.116)
3	negative	0.005	0.078	0.944	(-0.148,	0.159)	0.071	0.073	0.331	(-0.072,	0.214)
3	[neg.-min.]	-0.014	0.091	0.880	(-0.193,	0.165)	0.054	0.088	0.537	(-0.118,	0.227)
5	minimal	0.000	0.062	0.998	(-0.121,	0.121)	0.019	0.057	0.743	(-0.092,	0.129)
5	negative	0.010	0.089	0.914	(-0.165,	0.184)	0.105	0.084	0.213	(-0.060,	0.269)
5	[neg.-min.]	0.010	0.103	0.927	(-0.193,	0.212)	0.086	0.100	0.392	(-0.111,	0.283)
**Anxiety**											
1	minimal	0.026	0.062	0.668	(-0.095,	0.148)	-0.022	0.056	0.694	(-0.133,	0.088)
1	negative	0.042	0.086	0.622	(-0.126,	0.211)	0.069	0.082	0.397	(-0.091,	0.229)
1	[neg.-min.]	0.016	0.101	0.875	(-0.182,	0.213)	0.091	0.098	0.353	(-0.101,	0.284)
2	minimal	-0.025	0.067	0.714	(-0.156,	0.107)	-0.042	0.061	0.490	(-0.163,	0.078)
2	negative	-0.048	0.095	0.611	(-0.234,	0.138)	-0.065	0.090	0.469	(-0.242,	0.111)
2	[neg.-min.]	-0.024	0.111	0.831	(-0.241,	0.194)	-0.023	0.108	0.833	(-0.235,	0.189)
3	minimal	-0.016	0.065	0.806	(-0.144,	0.112)	-0.018	0.061	0.764	(-0.137,	0.101)
3	negative	-0.007	0.094	0.939	(-0.192,	0.177)	0.005	0.088	0.951	(-0.167,	0.177)
3	[neg.-min.]	0.009	0.110	0.936	(-0.207,	0.224)	0.024	0.106	0.823	(-0.184,	0.231)
5	minimal	0.057	0.071	0.426	(-0.083,	0.196)	0.041	0.065	0.529	(-0.087,	0.168)
5	negative	-0.089	0.102	0.384	(-0.290,	0.111)	-0.142	0.097	0.140	(-0.332,	0.047)
5	[neg.-min.]	-0.146	0.119	0.219	(-0.378,	0.087)	-0.183	0.116	0.113	(-0.410,	0.043)
**Happiness**											
1	minimal	-0.030	0.045	0.514	(-0.118,	0.059)	-0.031	0.041	0.447	(-0.112,	0.049)
1	negative	-0.032	0.063	0.611	(-0.155,	0.091)	0.005	0.060	0.938	(-0.112,	0.122)
1	[neg.-min.]	-0.002	0.074	0.974	(-0.147,	0.142)	0.036	0.072	0.616	(-0.105,	0.177)
2	minimal	0.021	0.046	0.646	(-0.069,	0.111)	0.032	0.042	0.450	(-0.051,	0.114)
2	negative	-0.067	0.065	0.300	(-0.194,	0.060)	-0.002	0.061	0.980	(-0.122,	0.119)
2	[neg.-min.]	-0.088	0.076	0.245	(-0.236,	0.060)	-0.033	0.074	0.652	(-0.178,	0.111)
3	minimal	-0.031	0.043	0.478	(-0.115,	0.054)	0.003	0.040	0.938	(-0.075,	0.081)
3	negative	-0.131	0.061	0.032	(-0.252,	-0.011)	-0.067	0.058	0.244	(-0.180,	0.046)
3	[neg.-min.]	-0.101	0.072	0.160	(-0.242,	0.040)	-0.070	0.069	0.312	(-0.206,	0.066)
5	minimal	-0.035	0.047	0.462	(-0.128,	0.058)	0.022	0.043	0.604	(-0.062,	0.107)
5	negative	0.026	0.068	0.698	(-0.106,	0.159)	-0.023	0.064	0.723	(-0.148,	0.102)
5	[neg.-min.]	0.061	0.079	0.438	(-0.093,	0.215)	-0.045	0.076	0.555	(-0.195,	0.105)
**Valued life**											
1	minimal	-0.046	0.052	0.376	(-0.148,	0.056)	0.036	0.047	0.451	(-0.057,	0.129)
1	negative	-0.094	0.072	0.194	(-0.236,	0.048)	0.045	0.069	0.511	(-0.090,	0.180)
1	[neg.-min.]	-0.048	0.085	0.571	(-0.215,	0.118)	0.009	0.083	0.910	(-0.153,	0.172)
2	minimal	0.100	0.052	0.056	(-0.002,	0.202)	0.067	0.048	0.162	(-0.027,	0.160)
2	negative	-0.186	0.073	0.011	(-0.330,	-0.042)	-0.083	0.070	0.236	(-0.219,	0.054)
2	[neg.-min.]	**-0.286**	0.086	0.001	(-0.454,	-0.117)	-0.149	0.084	0.075	(-0.313,	0.015)
3	minimal	0.022	0.048	0.652	(-0.073,	0.116)	0.078	0.045	0.080	(-0.009,	0.165)
3	negative	-0.058	0.069	0.402	(-0.193,	0.077)	0.035	0.065	0.587	(-0.092,	0.162)
3	[neg.-min.]	-0.080	0.081	0.324	(-0.238,	0.079)	-0.043	0.078	0.581	(-0.195,	0.110)
5	minimal	0.044	0.055	0.424	(-0.064,	0.152)	0.079	0.050	0.119	(-0.020,	0.178)
5	negative	-0.011	0.079	0.894	(-0.166,	0.145)	-0.046	0.075	0.541	(-0.192,	0.101)
5	[neg.-min.]	-0.054	0.092	0.554	(-0.235,	0.126)	-0.124	0.090	0.164	(-0.300,	0.051)

Note: Marginal effects were calculated from random intercept models fit to each outcome that include interaction terms between experimental conditions and an indicator for those negatively affected by the COVID-19 pandemic (vs. minimally effected). Estimates were allowed to vary across weeks. Differences between negative and minimal marginal effects and their associated significance tests were calculated using interaction terms. N = 1015. Respondents who reported being positively affected by the pandemic were excluded (N = 37). Models controlled for baseline measures of all outcomes as well as the effort respondents reported putting into performing behaviors. The prosocial acts condition was compared to both the neutral acts (control) and self-focused acts conditions, and effects are standardized mean differences. All coefficient tests are two-tailed. Differences in effects with *p* < 0.05 are bolded.

### Further exploratory analyses

As indicated previously, respondents varied in how much they complied with study instructions. This suggests the possibility that the non-effects observed in the pre-registered and planned exploratory analyses might be due to the inclusion of non-compliant respondents. To test this, we had to determine what qualified a respondent as “compliant”. We first examined if effects changed when restricting the sample to those who completed the majority of survey waves, which we operationalized as having completed at least 70%, 80% or 90% of the total number of surveys. In none of these cases did the pattern of effects differ substantively from the pre-registered analyses (see the replication files for full details).

**Fig 3:**
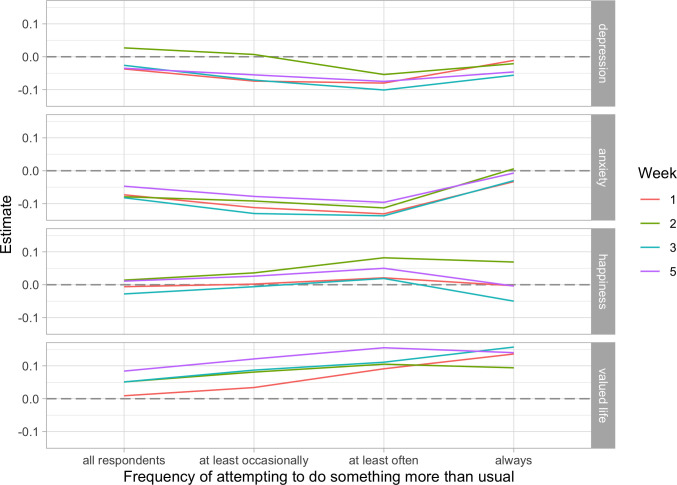
Estimates of the prosocial vs. neutral acts control effects on depression, anxiety, happiness, and valued life at different levels of self-reported effort.

**Fig 4:**
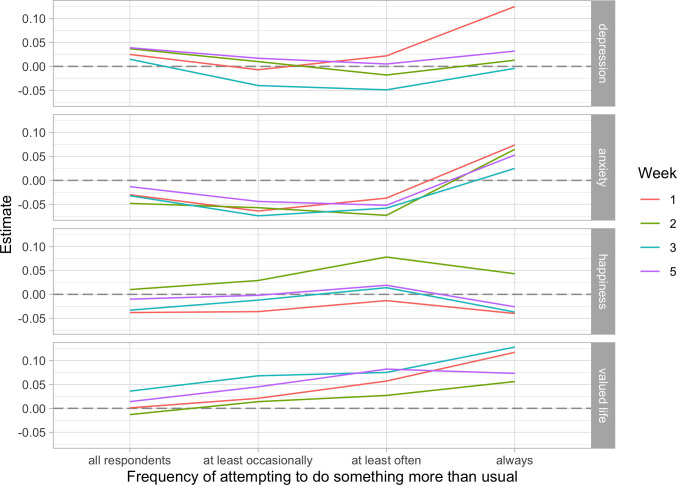
Estimates of the prosocial vs. self-focused effects on depression, anxiety, happiness, and valued life at different levels of self-reported effort.

We also examined whether results differed for those who reported putting more effort into the experiment. Those in the self-focused and prosocial conditions were instructed at baseline to perform acts that were “over and above” what they typically do, so we restricted our sample in turn to those who at least occasionally, often, or always tried to do something beyond what they normally do. We retained all respondents from the neutral acts control condition as they were not required to put it extra effort. To see how estimates changed, we plotted estimates for the full sample along with each of our restricted samples. Results for the prosocial vs. control effects are shown in Fig 3, and for prosocial vs. self-focused effects in Fig 4. Turning first to Fig 3, we see that in every case, restricting the sample to those who put in more effort moves estimates in the hypothesized directions—that is, towards lower levels of depression and anxiety, and higher levels of happiness and a sense that one’s life is valuable. This trend remains consistent until the sample is restricted to those who reported “always” trying to perform acts beyond what they normally do, at which point effects either plateau or reverse. The same patterns are evident in Fig 4.

The shift for those who always tried to go above and beyond their normal behavior might reflect an unexpected effect of the interventions (e.g., a reversal brought on by repeated forced compliance with experimental instructions, which could undermine a sense of autonomy [72]), or it might represent a selection effect of a particular subset of highly compliant individuals. Such individuals might have worse outcomes if high compliance is facilitated by characteristics that are associated with distress, such as perfectionism or low autonomy [72,90]. However, estimates for the “always” group are based on very few cases, and so we are hesitant to place too much weight on these interpretations (N_prosocial_ = 50; N_self-focused_ = 83).

Uncertainties about the most highly compliant aside, Figs 3 and 4 indicate that more compliant respondents generally responded as expected to our prosocial intervention. To test this formally, we re-estimated our analysis models using just those from the prosocial and self-focused conditions who reported often or always trying to do something more than what they usually do—we refer to these as “high effort” respondents—plus all respondents from the neutral acts control condition. This restriction reduced sample sizes to N = 131 in the prosocial condition and N = 208 in the self-focused condition, for a loss of 62% and 43% of the original sample size, respectively. Because estimates generally did not differ much across weeks, we constrained estimates for a given effect to be the same across weeks to increase statistical power. These models invariably fit the data better than models that allowed coefficients to vary by week (minimum differences in fit were: ΔAIC = -5.47, ΔBIC = -32.59, see the S7 Appendix). For comparison, we also fit equivalent models to the full sample.

Results are shown in Table 5. In the full sample, estimates were generally close to 0 and none were statistically significant. Once the sample was restricted to those who reported high effort, several effects aligned with our theoretical expectations. Compared to neutral acts controls, those who performed prosocial behavior reported a greater belief that their lives were valuable (β = 0.113, *p* = 0.005) and lower anxiety (β = -0.121, *p* = 0.017), consistent with hypotheses 2a and 4a. Effects on depression also trended in a beneficial direction but were not significant at the 0.05 level (β = -0.079, *p* = 0.089). Those in the prosocial condition did not differ significantly from those in the self-focused acts condition, failing to support hypotheses 1b-4b, though again the point estimates suggest a trend toward slightly better outcomes for those acting prosocially. As above, we also tested whether effects differed for those negatively affected by the pandemic. We found no significant differences (lowest *p*-value = 0.162; see S7 Appendix).

**Table 5:** Pooled effects of prosocial behavior on depression, anxiety, happiness, and valued life for full sample and respondents reporting high effort.

	Prosocial vs. Control					Prosocial vs. Self-Focused				
Outcome	Estimate	SE	p	95% CI		Estimate	SE	p	95% CI	
**Full sample**										
depression	0.003	0.033	0.931	(-0.062,	0.068)	0.050	0.033	0.123	(-0.014,	0.114)
anxiety	-0.043	0.038	0.259	(-0.117,	0.032)	-0.005	0.037	0.895	(-0.078,	0.068)
happiness	-0.007	0.028	0.804	(-0.062,	0.048)	-0.011	0.028	0.703	(-0.065,	0.044)
valued life	0.040	0.031	0.205	(-0.022,	0.102)	0.026	0.031	0.397	(-0.034,	0.087)
**High effort sample**										
depression	-0.079	0.046	0.089	(-0.170,	0.012)	-0.012	0.050	0.802	(-0.110,	0.085)
anxiety	**-0.121**	0.051	0.017	(-0.221,	-0.022)	-0.054	0.055	0.322	(-0.161,	0.053)
happiness	0.042	0.038	0.272	(-0.033,	0.116)	0.025	0.041	0.541	(-0.055,	0.105)
valued life	**0.113**	0.041	0.005	(0.033,	0.193)	0.061	0.044	0.163	(-0.025,	0.146)

Note: Estimates are coefficients from random intercept models fit to each outcome. Coefficients were constrained to be equal across all weeks of the study. Models controlled for baseline measures of all outcomes. Only “high effort” respondents who reported “often” or “always” trying to perform acts beyond what they normally do were included, plus all respondents from the neutral acts control condition (N = 679; N_prosocial_ = 131, N_self_ = 208, N_control_ = 340). The prosocial acts condition was compared to both the neutral acts (control) and self-focused acts conditions, and effects are presented as standardized mean differences. All coefficient tests are two-tailed. Effects with *p* < 0.05 are bolded.

Because outcomes were standardized, the effects presented in Table 5 are standardized mean differences that are roughly equivalent to Cohen’s *d*. The significant effects for anxiety (β = -0.121) and perceiving one’s life as valuable (β = 0.113) are about 40% smaller than the effect size of *d* = 0.19 anticipated by our power analysis, and roughly 60% smaller than the *d* = 0.28 effect found in a recent meta-analysis of prosocial behavior and well-being [17].

## Discussion

Our study was designed to overcome several limitations of research on the effects of prosocial behavior on emotions, in particular the use of small samples leading to low power to detect effects, and an overwhelming focus on positive affect and happiness outcomes [17]. We accordingly conducted a high-powered preregistered study comparing the effects of prosocial, self-focused, and affectively neutral behaviors on four outcomes: happiness, the sense that one’s life is valuable, depression, and anxiety. We hoped that our results would not only inform the literature on prosocial behavior, but also provide insight into whether prosocial actions could be used to safeguard mental health and promote emotional well-being amidst a global pandemic and its associated public health measures. To that end, we also assessed whether prosocial behavior had different effects for those who had been negatively affected by the COVID-19 pandemic.

Our pre-registered analyses failed to support any of our hypotheses. Those assigned to perform prosocial acts did not differ significantly in depression, anxiety, happiness, or the belief that their life had meaning and was valuable from those assigned to report their daily activities (hypotheses 1a-4a), nor from those who engaged in self-focused acts of personal gratification (hypotheses 1b-4b). Further, our planned exploratory analyses failed to detect meaningful differences in effects for those who had been negatively impacted by the pandemic compared to those who were relatively unaffected.

These non-findings are at odds with past work, which typically finds small effects of prosocial activity on a range of well-being and mental health outcomes. There are several possible reasons that our study failed to replicate these effects. One is that the hypothesized effects do not exist. While this strikes us as possible for some effects—particularly those related to less studied outcomes like depression and anxiety—we find it unlikely that this is true of *all* of the underlying effects given the presence of prosocial effects on indicators of emotional well-being in past meta-analyses and at least one high-powered replication [17,19,40]. Another possibility is that effects exist but are short-lived. In that case, we would have missed the effects by choosing to measure outcomes at a delay of several days. While we believe that prosocial acts might well have short term effects, existing evidence also suggests that effects can last for days or even weeks [e.g., 13,14]. We discuss this work further below. Yet another possibility is that problems with our study design prevented us from detecting effects. Design problems can never be ruled out completely, but we do not believe these to have been a major issue in this case given how closely this study mirrored elements of previous research, ranging from the multi-week design and the use of self-reported behaviors down to the wording of the experimental manipulations. These elements could likely be improved on—a point we return to momentarily—but given the ability of past studies to detect prosocial effects using similar designs, we do not think these features can fully explain our lack of significant findings.

What strikes us as more likely is that we simply did not adequately anticipate how to detect and adjust for non-compliance with study protocols. This interpretation is borne out by our unplanned exploratory analyses, which examined how various forms of non-compliance affected the results. Restricting the sample based on how many of the available survey respondents completed all survey waves did not alter the results, but restricting the sample based on self-reported effort did. At the start of the study respondents in the prosocial and self-focused conditions had been instructed to perform behaviors that were “over and above what you typically do.” Those who performed prosocial acts and reported at the end of the intervention that they often or always complied with these instructions showed a small reduction in anxiety, and a small increase in the belief that their lives were valuable.

Of course, restricting analyses to compliant respondents means that the observed effects will not generalize to non-compliant respondents if those respondents systematically differ from compliers in ways that matter for emotional well-being or mental health. We did not observe strong correlations between baseline measures of well-being and mental health and the amount of effort put into the intervention by those in the prosocial intervention condition (see replication materials for details), but it is possible that the compliant and non-compliant differed in ways we could not observe. Further, the loss of non-compliant respondents means that these analyses were underpowered. Post-hoc calculations using the obtained effect sizes for the prosocial intervention indicates that power to detect effects at the 0.05 level were roughly 0.28 (happiness), 0.42 (depression), 0.62 (anxiety), and 0.84 (valued life; see our replication materials for details). This suggests that prosocial acts *might* have real effects on both emotional well-being and mental health, but before drawing firm conclusions these results need to be replicated in an adequately powered study with stricter, pre-registered procedures for identifying and dealing with non-compliant respondents.

Assuming that these exploratory results hold up under further scrutiny, what are the implications? Perhaps the most important is that prosocial acts can provide a range of emotional benefits, including helping to reduce (or possibly prevent) anxiety. In the prosocial literature, anxiety has largely been studied in the context of interpersonal relationships, or else among those suffering from anxiety disorders [91–93]. To our knowledge, our study is one of the first to examine how prosocial acts can serve to reduce anxiety outside of the relationship context and for those not particularly suffering from abnormal levels of anxiety. Prosocial benefits might also extend to those suffering from depression, particularly those with more severe symptoms. For example, Schacter and Margolin found that adolescents with higher depressive symptoms saw greater increases in their daily mood compared to those without or with lower depressive symptoms) when they behaved prosocially [94]. Our analyses are consistent with the claim that prosocial acts can reduce depression but offer only weak support. The observed prosocial effect on depression was quite small, and its *p*-value was 0.089, just above our pre-registered alpha level of 0.05. It could instead be that those suffering from depression cannot generally enjoy the benefits of prosocial acts given that depression is typically characterized by negative self-views, feelings of unworthiness, and difficulty experiencing pleasure [94–96]. Determining if prosocial actions can relieve or prevent depressive symptoms is an important area for continued study.

It is worth reemphasizing that the effects we obtained are quite small; smaller, in fact, that those observed in past research [17,19]. It is unclear if this is due to sampling variability, an unobserved feature of our sample, or to the unique environment imposed by the COVID-19 pandemic (e.g., fewer in person contacts). It might also be that, despite our efforts to adopt best practices from prior studies on prosocial behavior, our intervention ended up being less effective than prior efforts. Regardless, it is clear that prosocial acts are not an emotional panacea. However, we believe they continue to have therapeutic potential for several reasons.

First, prosociality is flexible and cost effective. There are numerous ways to help others, many of which require nothing more than a little time and effort. This means that prosociality can be used by many people, and in many circumstances. For instance, we found no evidence that the positive effects of prosocial acts diminished for those who had been negatively impacted by the COVID-19 pandemic, which might suggest that the emotional effectiveness of prosocial acts are unaffected by social and economic circumstances of a person’s life. Of course, it might be that some people benefit more from prosocial acts than others, and/or that certain types of activities confer greater benefits than others. For instance, some research suggests that prosocial acts—if enacted willingly—foster emotional well-being by satisfying basic psychological needs such as relatedness, competence, or (possibly) morality [97,98]. If so, then we would expect individuals to reap greater emotional benefits when their behaviors more directly fulfil those needs, such as by helping a close vs. casual friend, or performing acts that they regard as particularly moral. Previous work also suggests that variety might be key because variation counteracts the tendency to acclimate to repeated experiences [99]. Our unplanned exploratory analyses are consistent with this idea—prosocial acts only yielded benefits for those who tried to do more than was typical for them. In short, many questions remain about how to maximize the emotional benefits of prosocial behavior, but existing evidence indicates that many people can experience some benefit with a relatively modest investment.

Another feature of prosociality effects on well-being is their longevity. Studies examining the longevity of prosocial interventions have shown that positive effects on various outcomes can last anywhere from several weeks to several months [10–15]. Our study is consistent with this work. In our unplanned exploratory analyses, we found models that constrained effects to be equal over time fit the data better than models that allowed effects to differ, suggesting that the beneficial effects of prosocial actions appeared early, persisted throughout the three weeks of the study, and lasted until the follow-up two weeks later. This suggests that prosocial acts might provide both short and long-term benefits. Further, these benefits arose even though respondents were only asked to perform prosocial acts for the first three days of each week. Respondents were also not asked to act prosocially at all between the end of the intervention and the follow-up, though many reported doing so at least occasionally. However, we found little evidence that effects at follow-up required respondents to continue regularly performing prosocial acts (see replication materials). This indicates that individuals can reap the rewards of prosocial behavior even if they engage in such acts only periodically.

Taken together, these considerations suggest that prosocial acts are valuable because they provide small, lasting benefits to emotional well-being and mental health, and that these benefits are likely available to many people who are navigating many types of personal circumstances.

The current study suggests a few avenues for future research. First, as noted above, it is important that our unplanned exploratory results be replicated, preferably using a pre-registered plan for dealing with study non-compliance. While we believe the choices that we made in identifying non-compliant respondents were sensible, the fact remains that they were post-hoc decisions that might have inadvertently affected the results we obtained. Relatedly, researchers should consider how they can increase compliance with study protocols. In our sample, only 38% of those asked to put in extra effort to perform prosocial behavior reported often or always doing so, a figure that rose to only 57% for those asked to engage in personally enjoyable acts. Willingness to put in extra effort might have been low given our use of a paid, online sample. It might also have been affected by our study design. For example, during the baseline survey we instructed respondents to put forth extra effort to enact prosocial behaviors but did not remind them of this requirement later in the study. More respondents might have followed these instructions if we had provided periodic reminders. Regardless of the reason, low compliance rates can drastically undermine statistical power to detect effects, so it is important for researchers to consider how compliance can be increased in future work.

Second, researchers should consider how to verify that respondents actually perform the behaviors they report. Consistent with previous studies, we asked participants to describe the behaviors they performed using daily “check-in” surveys. Further, we provided a relatively risk-free opportunity for respondents to inform us if they falsely reported behavior. At the end of the intervention—that is, at the end of week 3 but prior to the week 5 follow-up survey—we asked respondents the following: “For the last three weeks we’ve asked you to [CONDITION SPECIFIC PROMPT], and to report what you did to us. We realize that this could be challenging to do consistently, so we’d like to see now how you did. Please read the following options and select the one that best represents what you did.” Options ranged from “I never actually performed any of the behaviors I reported” to “I only reported behaviors that I had actually performed”. We further assured respondents that their responses would not affect their payment in any way. Only 1.7% of respondents admitted to ever falsely reporting behaviors. While we find this encouraging, the fact remains that we relied on the honest disclosure of participants. Moving forward, researchers may wish to use multiple sources of data to increase the reliability and accuracy of reports. For instance, researchers could ask others in a respondent’s close network to report the respondent’s behaviors and/or rate how much effort was put into completing those behaviors.

As seen in Figs 3 and 4, we found that beneficial effects of prosocial behavior increased as respondents put more effort into the trying to perform acts that were beyond what they normally do, but that this trend plateaued or reversed for those who *always* engaged in behavior beyond their regular routine. We suggested that this could be due to respondents feeling forced to perform acts, and/or to the possibility that highly compliant respondents are more likely to possess traits that can generate negative emotions, such as perfectionism. Determining whether these or other mechanisms account for this effect bears directly on how prosocial interventions should be designed. For instance, prosocial acts might yield optimal benefits only when encouraged rather than prescribed (i.e., autonomous vs. controlled behavior, [72]) and when failure to engage in such acts is normalized. We encourage researchers to begin untangling these processes. Given that in our study these respondents only made of 14.4% of respondent in the prosocial condition, this will likely require recruiting large (or targeted) samples to capture a greater number of highly compliant respondents.

Third, future work should seek to replicate our results in a variety of populations. Past work has found emotionally beneficial effects of prosocial behavior in samples of students, community members, online respondents, clinical samples, corporate workers, and children [10,13–15,22,26,64]. However, some populations (students, online respondents) and some outcomes (happiness, positive affect) have been more thoroughly studied than others. Thus, while we might have a *general* sense that prosocial acts yield emotional benefits for a variety of people, *specific* claims about prosocial effects on particular outcomes (e.g., anxiety, sense of meaning) and in particular populations (e.g., non-student, offline adults) are often based on little direct evidence.

Finally, future work could profitably be directed toward determining which factors explain the benefits of prosocial activity. Previous work suggests that prosocial acts may provide emotional rewards when they are undertaken willingly, satisfy basic psychological needs, and/or are varied to prevent acclimation. Here we add two other possibilities. First, prosocial effects might rely heavily on memory. Previous work has shown that recall can powerfully evoke emotions, and that recalling behaviors might have comparable effects to actually performing those behaviors [100,101]. Such strong memory effects could explain how prosocial acts can affect mental health and emotional well-being days or weeks later. It also suggests that individuals might reap some emotional benefits from performing even a modest number of prosocial acts, so long as they can continue to recall them clearly. Second, the *reason* for engaging in prosocial acts might moderate their effects. Previous studies suggest that motivations underlying altruistic behavior can affect the benefits that they provide [e.g., 31]. For example, a review of social motivation found that those with an “other orientation” (that is, those who engaged in prosocial behaviors with unselfish motives) experienced a range of mental health benefits, including increased psychological well-being [102]. If the same holds true for mental health, then this presents a paradox for researchers: if participants engage in routine prosocial behaviors, they may not reap the emotional benefits (as our study suggests). However, if researchers over-emphasize the benefits to participants to increase engagement, participants also may not reap the rewards if they have been driven to act based on selfish motives. Future research should seek to identify how robust prosocial effects on emotions are to variations in individual motives for engaging in prosocial acts.

## Conclusion

Past work suggests that prosocial behavior might provide a low-cost, easy to implement method of increasing positive emotions and boosting mental health. However, many questions remain about what outcomes prosocial acts influence, and how strong their effects are. The pre-registered analyses reported here failed to detect significant effects for any outcome, both among those who were minimally and negatively affected by the COVID-19 pandemic. However, unplanned exploratory analyses that removed low-effort respondents found small beneficial effects on anxiety and the sense that one’s life if valuable, suggesting that prosocial acts might have some therapeutic potential. Determining the scope of prosocial benefits will require additional high-powered studies with clear, pre-registered plans for managing study compliance.

## Supporting information

S1 Appendix Recruitment materials for Amazon’s Mechanical Turk. (DOCX)

S2 Appendix Prompts for prosocial intervention task. (DOCX)

S3 Appendix Questionnaire for baseline survey. (DOCX)

S4 Appendix Questionnaire for daily surveys. (DOCX)

S5 Appendix Questionnaire for end of week surveys. (DOCX)

S6 Appendix Sample description by experimental condition. (DOCX)

S7 Appendix Additional results. (DOCX)

S8 Appendix Examples of behaviors reported by experimental condition. (DOCX)

S1 File (PDF)
