# Supplementary material for: Using prosocial behavior to safeguard mental health and foster emotional well-being during the COVID-19 pandemic: A registered report of a randomized trial
Source: PLoS One. 2022 Jul 28;17(7):e0272152. doi: 10.1371/journal.pone.0272152 (PMC9333215; doi:10.1371/journal.pone.0272152)
Supplement: S3 Appendix — (DOCX) [file pone.0272152.s003.docx]

**S3 Appendix. Questionnaire for Baseline Survey**

**Survey text for VPS screening process** (see Supplemental Information from Kennedy et al. 2020)

[IF COUNTRY OTHER THAN CANADA OR UNITED STATES IS DETECTED, OR VPN USE IS DETECTED]

Our system has detected that you are trying to complete this survey from a location outside of the United States or Canada, or that you are using a Virtual Private Server (VPS) or proxy to mask your country location. VPS use has caused a number of problems with data quality (see <https://goo.gl/WD6QD4>).

Because of this, we cannot let you participate in this study. If you are located in the United States or Canada, consider turning off your VPS the next time you participate in a survey-based HIT. If you are outside of the United States or Canada, we apologize but this study is directed only towards residents of those countries (as per our ethics protocol).

Thank you for your interest in our study.

If you believe you have received this message in error, please report it to the requester for this study and enter your MTurk Worker ID below.

[TEXT BOX]

[NOTE: If our review suggests that there is a reasonable chance that the respondent is in the United States or Canada, we will pay them compensation for the lost survey, though they will be unable to continue on in study. We will err on the side of generosity. Relevant review criteria will be lookup of their IP address from one or more additional IP lookup services, as well as any information they provide in their email to the research team]

[IF WE ARE UNABLE TO DETERMINE LOCATION OR VPN USE]

For some reason we were unable to verify your country location.

Once you click Next, you will be taken to the survey (and certifying that you are taking this survey from the United States or Canada, and not using a VPS). We will be checking locations manually for those who reach this point and you will be contacted if this check identifies you as violating these requirements.

[NOTE: We will try to confirm the location of the IP address from one or other IP lookup services. We will pay the respondent in either case. If we are able to verify that the IP address comes from the United States or Canada, the respondent will be retained in the study. Otherwise, the respondent will be removed from the study sample]

[RANDOMIZE THE ORDER OF ALL BLOCKS OF QUESTIONS BELOW AND THE ORDER OF ALL QUESTIONS WITHIN BLOCKS UNLESS OTHERWISE INDICATED]

*Emotional Well-Being: Subjective Happiness Scale (see Aknin et al 2013; Lyubomirsky and Lepper 1999)*

For each of the following statements and/or questions, please select the point on the scale that you feel is most appropriate in describing you.

SHS1. In general, I consider myself

1. not a very happy person

2.

3.

4.

5.

6.

7. a very happy person

SHS2. Compared to most of my peers, I consider myself:

1. less happy

2.

3.

4.

5.

6.

7. more happy

SHS3. Some people are generally very happy. They enjoy life regardless of what is going on, getting the most out of everything. To what extend does this characterization describe you?

1. not at all

2.

3.

4.

5.

6.

7. a great deal

SHS4. Some people are generally not very happy. Although they are not depressed, they never seem as happy as they might be. To what extent does this characterization describe you?

1. not at all

2.

3.

4.

5.

6.

7. a great deal

*Sense of Purpose and Meaning* (from Morgan and Farsides 2009: valued life is a sub-scale of the 23-item meaning in life scale)

Rate your agreement or disagreement with the following statements.

Valued Life

VL1. My life is worthwhile.

VL2. My life is significant.

VL3. I really value my life.

VL4. I hold my own life in high regard.

-3. Strongly disagree

-2. Disagree

-1. Slightly disagree

0. Neither agree nor disagree

1. Slightly agree

2. Agree

3. Strongly agree

*Mental Health*

*Depression (Modified/Shortened CES-D Scale) and Anxiety (HADS-A)*

Below is a series of statements about how you might have felt or behaved during the past week. For each one, indicate how often during the past week you felt like this.

Don't take too long over your replies; your immediate reaction to each item will probably be more accurate than a long thought-out response.

| CESD1 | During the past week I felt depressed |
| --- | --- |
| CESD2 | During the past week I felt that everything I did was an effort |
| CESD3 | During the past week my sleep was restless |
| CESD4 | During the past week I was happy |
| CESD5 | During the past week I felt lonely |
| CESD6 | During the past week I enjoyed life |
| CESD7 | During the past week I felt sad |
| CESD8 | During the past week I could not get "going" |
| HADS1 | During the past week I felt tense or "wound up." |
| HADS2 | During the past week I got a frightened feeling as if something awful was about to happen. |
| HADS3 | During the past week worrying thoughts went through my mind. |
| HADS4 | During the past week I got a frightened feeling like butterflies in my stomach. |
| HADS5 | During the past week I felt restless as if I had to be on the move. |
| HADS6 | During the past week I had a sudden feeling of panic. |
| HADS7 | During the past week I could sit at ease and feel relaxed. |

1. rarely or none of the time
2. some of the time
3. a moderate amount of the time
4. most or all of the time

[END BLOCK RANDOMIZATION – THE FOLLOWING QUESTIONS SHOULD BE PRESENTED IN THE ORDER SHOWN]

*COVID-19 Impact*

How has your _______ been affected by the COVID-19 pandemic?

| COV_SOCIAL | social life (non-family) |
| --- | --- |
| COV_FAM | family life |
| COV_FIN | financial situation |
| COV_EMP | employment |
| COV_LEIS | leisure time |

1. Very negatively affected
2. Negatively affected
3. Somewhat negatively affected
4. Not affected
5. Somewhat positively affected
6. Positively affected
7. Very positively affected

*Demographics*

D1. What is your gender?

1. Male
2. Female
3. Other (please specify)

D2. How old are you (in years)? [responses from 18 through 75+]

D3. What is the highest level of education that you have completed?

1. Less than high school
2. High school diploma or GED
3. Some college
4. Associate’s degree
5. Bachelor’s degree
6. Graduate or professional degree

D4. In which [state/province] do you currently reside? [list of all states, or provinces if Canadian data]

D5. Which racial category best describes you? [select all that apply]

1. White
2. Black
3. Hispanic
4. Asian
5. Other (please specify) [TEXT ENTRY]

D6. What is your marital status?

1. Never been married
2. Married
3. Living with someone (not married)
4. Separated
5. Divorced
6. Widowed

D7. Generally speaking, do you think of yourself as a:

1. Protestant
2. Evangelical Protestant
3. Catholic
4. Jew
5. Mormon/Latter-day Saint
6. Muslim
7. Not religious
8. Other (please specify) [text box]

D8. During the past year, about how often did you attend religious services?

1. Never
2. A few times
3. About once a month
4. 2 to 3 times a month
5. Once a week
6. More than once a week

D9. Religiously, I consider myself:

1. Very religious
2. Religious
3. Somewhat religious
4. Religious in name only
5. Not religious
6. Anti-religious

D10. About how much money do you, personally, *typically* make each year?

1. $0
2. $1-$20,000
3. $20,001 - $40,000
4. $40,001 - $60,000
5. $60,001 - $80,000
6. $80,001 - $100,000
7. $100,001 - $120,000
8. $120,001 - $140,000
9. More than $140,000

D11. Altogether, about how much money do people living in your household *typically* make each year (including you)?

1. $0
2. $1-$20,000
3. $20,001 - $40,000
4. $40,001 - $60,000
5. $60,001 - $80,000
6. $80,001 - $100,000
7. $100,001 - $120,000
8. $120,001 - $140,000
9. More than $140,000

*Prosocial Intervention Task*

[THE INTERVENTION IS SHOWN IN S2 APPENDIX]

*Conclusion and Opt-Out*

That’s it! Thanks for taking today’s survey.

This survey was the first part of a three-week study. Over the next three weeks, we will send you several short surveys each week, plus longer surveys at the end of each week. All surveys will be administered through Mechanical Turk, and you’ll be paid for each survey you complete. In total, you can earn up to $16. We’ll send you an email reminder each day that there is a survey ready for you.

If you agree to continue on in this study, please select **Yes** below. If you no longer wish to take part in this study, please select **No** below. You will still be paid for your responses today if you choose not to continue.

OPTOUT: Do you wish to continue taking part in this study?

0=No

1=Yes

[IF OPTOUT=YES]

Great! We’re glad to have you. We’ll send you the first daily survey tomorrow.

[IF OPTOUT=NO]

You have opted out of this study.

FR. Is there anything else you’d like to tell us? (this question is optional)

[TEXT FIELD]

Please enter the survey code to receive payment for participating in today’s survey.

*NOTE: This questionnaire is an edited version of the full questionnaire that focuses on just those parts of the project that are relevant to the preregistered study. Other measures designed for subsequent exploratory analyses are not included here.*
