# Supplementary material for: Using prosocial behavior to safeguard mental health and foster emotional well-being during the COVID-19 pandemic: A registered report of a randomized trial
Source: PLoS One. 2022 Jul 28;17(7):e0272152. doi: 10.1371/journal.pone.0272152 (PMC9333215; doi:10.1371/journal.pone.0272152)
Supplement: S6 Appendix — (DOCX) [file pone.0272152.s006.docx]

**S6 Appendix. Sample Description by Experimental Condition**

|  | **Control** | | | | **Self-Focused** | | | | **Prosocial** | | | |
| --- | --- | --- | --- | --- | --- | --- | --- | --- | --- | --- | --- | --- |
|  | **Mean** | **S.D.** | **Min** | **Max** | **Mean** | **S.D.** | **Min** | **Max** | **Mean** | **S.D.** | **Min** | **Max** |
| *Baseline Outcomes* |  |  |  |  |  |  |  |  |  |  |  |  |
| Depression | 8.99 | 5.73 | 2 | 26 | 8.72 | 5.78 | 2 | 26 | 9.00 | 5.87 | 2 | 26 |
| Anxiety | 5.69 | 4.22 | 1 | 20 | 5.80 | 4.49 | 1 | 22 | 5.90 | 4.47 | 1 | 22 |
| Happiness | 4.48 | 1.54 | 1 | 7 | 4.54 | 1.46 | 1 | 7 | 4.41 | 1.55 | 1 | 7 |
| Valued life | 1.62 | 1.43 | -3 | 3 | 1.65 | 1.28 | -3 | 3 | 1.60 | 1.37 | -3 | 3 |
| Age | 45.78 | 12.86 | 18 | 79 | 46.18 | 12.98 | 24 | 78 | 46.88 | 12.75 | 18 | 79 |
| *Gender* |  |  |  |  |  |  |  |  |  |  |  |  |
| Male | 0.52 | 0.50 | 0 | 1 | 0.48 | 0.50 | 0 | 1 | 0.50 | 0.50 | 0 | 1 |
| Female | 0.47 | 0.50 | 0 | 1 | 0.52 | 0.50 | 0 | 1 | 0.49 | 0.50 | 0 | 1 |
| Other | < 0.01 | 0.05 | 0 | 1 | < 0.01 | 0.05 | 0 | 1 | 0.01 | 0.11 | 0 | 1 |
| *Education* |  |  |  |  |  |  |  |  |  |  |  |  |
| High school or less | 0.11 | 0.31 | 0 | 1 | 0.08 | 0.28 | 0 | 1 | 0.10 | 0.30 | 0 | 1 |
| Some college | 0.18 | 0.38 | 0 | 1 | 0.18 | 0.38 | 0 | 1 | 0.14 | 0.35 | 0 | 1 |
| Associates | 0.10 | 0.30 | 0 | 1 | 0.12 | 0.32 | 0 | 1 | 0.10 | 0.30 | 0 | 1 |
| BA | 0.41 | 0.49 | 0 | 1 | 0.42 | 0.49 | 0 | 1 | 0.44 | 0.50 | 0 | 1 |
| Graduate/Professional | 0.21 | 0.41 | 0 | 1 | 0.21 | 0.41 | 0 | 1 | 0.21 | 0.41 | 0 | 1 |
| *Race* |  |  |  |  |  |  |  |  |  |  |  |  |
| White | 0.81 | 0.40 | 0 | 1 | 0.81 | 0.39 | 0 | 1 | 0.77 | 0.42 | 0 | 1 |
| Black | 0.06 | 0.24 | 0 | 1 | 0.03 | 0.16 | 0 | 1 | 0.04 | 0.20 | 0 | 1 |
| Hispanic | 0.02 | 0.13 | 0 | 1 | 0.02 | 0.15 | 0 | 1 | 0.04 | 0.19 | 0 | 1 |
| Asian | 0.08 | 0.27 | 0 | 1 | 0.09 | 0.29 | 0 | 1 | 0.10 | 0.30 | 0 | 1 |
| Other/Mixed | 0.04 | 0.18 | 0 | 1 | 0.04 | 0.21 | 0 | 1 | 0.05 | 0.22 | 0 | 1 |
