# Supplementary material for: Using prosocial behavior to safeguard mental health and foster emotional well-being during the COVID-19 pandemic: A registered report of a randomized trial
Source: PLoS One. 2022 Jul 28;17(7):e0272152. doi: 10.1371/journal.pone.0272152 (PMC9333215; doi:10.1371/journal.pone.0272152)
Supplement: S7 Appendix — (DOCX) [file pone.0272152.s007.docx]

**S7 Appendix. Additional Results**

**Table S7.1: Differences in BIC and AIC for Models that Pool or Do Not Pool Estimates Across Weeks**

| **Outcome** | **Not Pooled** | **Pooled** | **Difference** |
| --- | --- | --- | --- |
| **BIC** | | | |
| depression | 10697.04 | 10661.13 | -35.91 |
| anxiety | 11447.65 | 11409.90 | -37.75 |
| happiness | 9109.65 | 9077.06 | -32.59 |
| valued life | 9891.87 | 9856.30 | -35.57 |
| **AIC** | | | |
| depression | 10425.80 | 10417.02 | -8.78 |
| anxiety | 11176.41 | 11165.78 | -10.63 |
| happiness | 8838.42 | 8832.95 | -5.47 |
| valued life | 9620.63 | 9612.19 | -8.44 |

Note: Negative difference scores indicate better fit for the pooled model.

**Table S7.2:** **Marginal Effects of Prosocial Behavior for Groups Minimally and Negatively Affected by the COVID-19 Pandemic for Respondents Reporting High Effort**

|  |  | **Prosocial vs. Control** | | | | |  | **Prosocial vs. Self-Focused** | | | | |
| --- | --- | --- | --- | --- | --- | --- | --- | --- | --- | --- | --- | --- |
| **COVID**  **impact** |  | **Estimate** | **SE** | **p** | **95% CI** | |  | **Estimate** | **SE** | **p** | **95% CI** | |
| **Depression** | | | | | | | | | | | | |
| minimal |  | -0.031 | 0.057 | 0.586 | (-0.143, | 0.081) |  | 0.008 | 0.061 | 0.895 | (-0.111, | 0.127) |
| negative |  | **-0.168** | 0.079 | 0.035 | (-0.324, | -0.012) |  | -0.067 | 0.087 | 0.441 | (-0.237, | 0.103) |
| [neg.-min.] |  | -0.137 | 0.098 | 0.162 | (-0.328, | 0.055) |  | -0.075 | 0.106 | 0.480 | (-0.282, | 0.133) |
| **Anxiety** | | | | | | | | | | | | |
| minimal |  | -0.062 | 0.063 | 0.331 | (-0.186, | 0.063) |  | -0.021 | 0.067 | 0.752 | (-0.153, | 0.111) |
| negative |  | **-0.192** | 0.088 | 0.029 | (-0.364, | -0.019) |  | -0.111 | 0.096 | 0.247 | (-0.299, | 0.077) |
| [neg.-min.] |  | -0.130 | 0.108 | 0.229 | (-0.342, | 0.082) |  | -0.090 | 0.117 | 0.444 | (-0.320, | 0.140) |
| **Happiness** | | | | | | | | | | | | |
| minimal |  | 0.049 | 0.048 | 0.307 | (-0.045, | 0.144) |  | 0.025 | 0.051 | 0.628 | (-0.076, | 0.125) |
| negative |  | 0.028 | 0.067 | 0.676 | (-0.103, | 0.159) |  | 0.026 | 0.073 | 0.721 | (-0.117, | 0.170) |
| [neg.-min.] |  | -0.021 | 0.082 | 0.796 | (-0.183, | 0.140) |  | 0.001 | 0.089 | 0.989 | (-0.174, | 0.177) |
| **Valued Life** | | | | | | | | | | | | |
| minimal |  | **0.112** | 0.051 | 0.027 | (0.013, | 0.212) |  | 0.066 | 0.054 | 0.224 | (-0.040, | 0.171) |
| negative |  | 0.089 | 0.070 | 0.208 | (-0.049, | 0.227) |  | 0.067 | 0.077 | 0.385 | (-0.084, | 0.217) |
| [neg.-min.] |  | -0.024 | 0.087 | 0.785 | (-0.194, | 0.146) |  | 0.001 | 0.094 | 0.990 | (-0.183, | 0.185) |

Note: Marginal effects were calculated from random intercept models fit to each outcome that include interaction terms between experimental conditions and an indicator for those negatively affected by the COVID-19 pandemic (vs. minimally effected). Models controlled for baseline measures of all outcomes. Estimates were constrained to be equal across all weeks of the study. Differences between negative and minimal marginal effects and their associated significance tests were calculated using interaction terms. Only “high effort” respondents who reported “often” or “always” trying to perform acts beyond what they normally do were included, plus all respondents from the neutral acts control condition. Further, respondents who reported being positively affected by the pandemic were excluded. N = 653. The prosocial acts condition was compared to both the neutral acts (control) and self-focused acts conditions, and effects are presented as standardized mean differences. All coefficient tests are two-tailed. Effects with *p* < 0.05 are bolded.
