# Supplementary material for: Using prosocial behavior to safeguard mental health and foster emotional well-being during the COVID-19 pandemic: A registered report of a randomized trial
Source: PLoS One. 2022 Jul 28;17(7):e0272152. doi: 10.1371/journal.pone.0272152 (PMC9333215; doi:10.1371/journal.pone.0272152)
Supplement: S8 Appendix — (DOCX) [file pone.0272152.s008.docx]

**S8 Appendix. Examples of Behaviors Reported by Experimental Condition**

The table below shows the first three behaviors reported on the first day of the intervention in each experimental condition. All other reported behaviors can be found in the study data file.

| Condition | Prompt |
| --- | --- |
| 1. Neutral control | 1. I took my son to the doctor this morning. He woke up this morning feeling bad and had a mild fever. With Covid-19 being out there these days, I figured it was best to get him to the doctor as soon as possible. The doctor checked everything out and even gave him a Covid-19 test, which luckily came back negative. He just stated that he has a case of the flu and gave him a shot and a couple of prescriptions. 2. Today, I went to get groceries for the week. I had to go to two grocery stores, since one did not have an item I was looking for. I went around 11am. 3. I took my son to baseball practice. |
| 2. Self-focused | 1. I listened to my audio book, for a few hours and learned some new things 2. Today to treat myself, I tuned everyone and everything out and took a long hot bath. It’s been years and I felt as if it was well deserved. 3. I had a bowl of ice cream last night. No bubble bath but a nice long shower. No time for a walk today. |
| 3. Prosocial intervention | 1. I took my granddaughter to eat at her favorite restaurant 2. I let my spouse sleep late and let the dogs out this morning 3. I spent approximately 30 minutes on Facetime assisting my 12 year old nephew with his science homework. My sister (his mom) did not know how to help him with the math portion of his physics assignment. Having taken physics in college, I was able to explain the concepts to him and help him complete him homework successfully. |
