# Supplementary material for: Using prosocial behavior to safeguard mental health and foster emotional well-being during the COVID-19 pandemic: A registered report of a randomized trial
Source: PLoS One. 2022 Jul 28;17(7):e0272152. doi: 10.1371/journal.pone.0272152 (PMC9333215; doi:10.1371/journal.pone.0272152)
Supplement: S1 File — (PDF) [file pone.0272152.s009.pdf]

ClinicalTrials.gov Protocol Registration and Results System (PRS) Receipt
Release Date: May 25, 2021

ClinicalTrials.gov ID: NCT04517006

Study Identification

Unique Protocol ID: 00039312

Brief Title: Prosocial Behavior Can Safeguard Mental Health and Foster Emotional Well-being During the COVID-19 Pandemic

Official Title: Prosocial Behavior Can Safeguard Mental Health and Foster Emotional Well-being During the COVID-19 Pandemic: A Randomized Clinical Trial

Secondary IDs:

Study Status

Record Verification: May 2021

Overall Status: Completed

Study Start: January 31, 2021 [Actual]

Primary Completion: April 4, 2021 [Actual]

Study Completion: April 4, 2021 [Actual]

Sponsor/Collaborators

Sponsor: University of Toronto

Responsible Party: Principal Investigator
Investigator: Andrew Miles [amiles]
Official Title: Assistant Professor
Affiliation: University of Toronto

Collaborators:

Oversight

U.S. FDA-regulated Drug: No

U.S. FDA-regulated Device: No

U.S. FDA IND/IDE: No

Human Subjects Review: Board Status: Approved
Approval Number: 00039312
Board Name: University of Toronto Research Ethics Board
Board Affiliation: University of Toronto
Phone: (416) 946-3389
Email: rachel.zand@utoronto.ca
Address:

McMurrich Building, 12 Queen's Park Crescent West, 2nd Floor, Toronto, ON M5S 1S8

Data Monitoring: No

FDA Regulated Intervention: No

Study Description

Brief Summary: The COVID-19 pandemic and the accompanying lockdown measures have made mental health a pressing public health concern. Acts that focus on benefiting others—known as prosocial behaviors—offer one promising intervention that is both flexible and low cost. However, neither the range of emotional states prosocial acts impact nor the size of those effects is currently clear, both of which directly influence its attractiveness as a treatment option. Using a large online sample from Canada and the United States, the investigators will examine the effect of a three-week prosocial intervention on two indicators of emotional well-being (happiness and the belief that one's life is valuable) and mental health (anxiety and depression). Respondents will be randomly assigned to perform prosocial, self-focused, or neutral behaviors each week. Two weeks after the intervention, a final survey will assess whether the intervention has a lasting effect on mental health and emotional well-being. The results will illuminate whether prosocial interventions are a viable approach to addressing mental health needs during the current COVID-19 pandemic, as well for those who face emotional challenges during normal times.

Detailed Description: This study is intended to be published as a registered report. The details of the study design are likely to change somewhat during the review process. However, below is the current plan:

The investigators will examine the effects of prosocial behavior using a 3-week experimental intervention, followed by a follow-up assessment at 5 weeks. At baseline the research team will measure participants' emotional well-being and mental health. Emotional well-being will be assessed using happiness and feeling that one's life is valuable, which is a facet of sense of meaning in life. Mental health will be measured as depression and anxiety. At the end of the baseline survey the researchers will randomly assign participants to one of three experimental conditions (between-subjects design). In each condition, respondents will be asked to perform certain types of behaviors for the first three days of each week, as detailed later in this protocol.

Respondents will be contacted for the first three days each week and asked to report what they did. At the end of weeks 1, 2, and 3 they will complete a longer survey that repeats the same measures of emotional well-being and mental health used at baseline. At this point the intervention will be complete. The investigators will recontact respondent two weeks later (at the end of week 5) to assess whether the intervention has a lasting effect on mental health and emotional well-being. This final survey will also include a measure of whether or not respondents continued their assigned behaviors following the end of the intervention. This measure might be useful for explaining any lasting effects in exploratory analyses.

The expected effect size is $d = 0.19$. The research team will assess experimental effects using multiple linear regression. The models will include indicator variables for experimental conditions as well as controls for baseline levels of happiness, valued life, depression, and anxiety. These baseline measures should be unrelated to experimental conditions (which will be randomly assigned) so including them will increase the efficiency of estimates. Under the assumption that these variables will account for at least 50% of the variance in the outcomes, the sample size needed to detect an effect of size $d = 0.19$ with 95% power is $N = 357$ or approximately 360 per condition. The

expected attrition rate is 30% after baseline which means that the investigators will sample 360/0.7 = 514 per condition.

The research team will post the baseline survey in two batches. The first batch will contain half the sample (N = 771), and will be used to gauge the attrition rate (see data exclusion criteria). The sample size of the second batch will be adjusted as needed to try and capture the desired number of respondents per experimental condition. This will lower the size of the second batch if attrition rates are lower than expected or increase the size of the second batch if attrition rates are higher than expected. If a sufficient sample size has not been obtained at this point, the investigators will repeat the process until at least 350 respondents in each experimental condition are recruited. To be clear, the stopping rule for recruiting sample participants does not require estimating any of the effects of interest in the study (i.e., it does not depend on the anticipated effect size). The only factor is whether a sufficient number of respondents have been recruited for each experimental condition.

Obtaining a sufficient per-condition sample size might take several days. The researchers plan to begin sampling on a Sunday and continue sampling (if needed) on Monday. This means respondents will finish their first three days of the study on either Wednesday or Thursday. In either case, the investigators will distribute the end of week 1 survey the following Sunday, which will then put both groups of respondents on the same schedule for the remainder of the study. The researchers will try to recruit the full sample during this initial recruitment phase, as this will simplify administering the study. However, if initial sampling does not yield a sufficient sample size, the researchers will repeat the sampling procedure each week until a sufficient number of respondents is obtained. In this case, the study will be administered to different “cohorts” of respondents spaced a week apart.

Conditions

Conditions: Happiness
Anxiety
Depression
Morality

Keywords:

Study Design

Study Type: Interventional

Primary Purpose: Supportive Care

Study Phase: N/A

Interventional Study Model: Factorial Assignment

Number of Arms: 3

Masking: None (Open Label)

Allocation: Randomized

Enrollment: 1328 [Actual]

Arms and Interventions

Arms	Assigned Interventions
No Intervention: Track activities	

Arms	Assigned Interventions
For three weeks research subjects will be keeping track of the things they do without altering their routine in any way.	
Experimental: Self-focused acts<br>For three weeks research subjects will be "treating themselves" by doing things that they enjoy. These acts don't have to be large or costly, but they should be over and above what they typically do. They are asked to do one (or more) things they enjoy each day for the first three days of each week and report them.	Behavioral: Self-focused acts<br>Completion of daily acts for personal enjoyment.
Experimental: Prosocial acts<br>For three weeks research subjects are asked to perform acts of kindness, meaning behaviors that benefit someone else and are over and above what they typically do (i.e., they are not expected of them). These acts should also involve some sacrifice by them (e.g., in effort, energy, time, or money) and be completed for the first three days of each week.	Behavioral: Prosocial acts<br>Completion of daily acts for the benefit of others.

Outcome Measures

Primary Outcome Measure:

1. Change in happiness from baseline using the subjective happiness scale
   The Subjective Happiness Scale consists of four items: (1) "In general, I consider myself _______." Responses options run from 1 = "not a very happy person" to 7= "a very happy person." (2) "Compared to most of my peers, I consider myself ________" with response options from 1 = "less happy" to 7 = "more happy." (3) "Some people are generally very happy. They enjoy life regardless of what is going on, getting the most out of everything. To what extent does this characterization describe you?" (1 = "not at all" to 7 = "a great deal"). (4) "Some people are generally not very happy. Although they are not depressed, they never seem as happy as they might be. To what extent does this characterization describe you?" (1= "not at all" to 7= "a great deal"; reverse coded) These four items will be averaged into a scale ranging from 1-7, where higher scores indicate greater subjective happiness.

   [Time Frame: baseline, end of weeks 1, 2, 3, and 5]
2. Change in valued life assessment from baseline using 4-item scale
   Respondents' individual perceptions of whether their life has value will be measured using the valued life subscale developed by Morgan and Farsides. This measure will consist of the average of the following four items: (1) "My life is worthwhile," (2) "My life is significant," (3) I really value my life," and (4) I hold my own life in high regard." In each instance, response options will run from -3 = "strongly disagree" to 3 = "strongly agree".

   [Time Frame: baseline, end of weeks 1, 2, 3, and 5]
3. Change in depression from baseline using the CESD (8-item)
   Depression will be measured using the well-established 8-item short-form of the Centre for Epidemiological Studies-Depression Scale (CES-D). Respondents will report how often in the past week they (1) felt depressed, (2) felt that everything was an effort, (3) felt that sleep was restless, (4) felt happy (reverse coded), (5) enjoyed life (reverse coded), (6) felt lonely, (7) felt sad, and (8) could not get going. Responses will be scored where 0 = "rarely or none of the time," 1 = "some of the time," 2 = "a moderate amount of time," and 3 = "most or all of the time."

   [Time Frame: baseline, end of weeks 1, 2, 3, and 5]
4. Change in anxiety from baseline using the HADS-A
   Anxiety will be measured with the Hospital Anxiety and Depression Scale—Anxiety (HADS-A), which is both commonly used and well-validated. This is a 7-item scales that asks respondents how often in the past week they: (1) felt tense or wound up, (2) got a frightened feeling as if something awful was about to happen, (3) had worrying thoughts go through their mind, (4) got a frightened feeling like butterflies in the stomach, (5) felt restless as if they had to be on the move, (6) had a sudden feeling of panic, and (7) could sit at ease and feel relaxed. To ensure consistency with our measure of depression, responses will be coded where 0 = "rarely or none of the time," 1 = "some of the time," 2 = "a moderate amount of time," and 3 = "most or all of the time."

[Time Frame: baseline, end of weeks 1, 2, 3, and 5]

Eligibility

Minimum Age: 18 Years

Maximum Age:

Sex: All

Gender Based: No

Accepts Healthy Volunteers: Yes

Criteria: Inclusion Criteria:

- Canadian and American respondents from the Amazon Mechanical Turk

Exclusion Criteria:

Respondents will be excluded from the study if any of the following apply:

1. A respondent completes the baseline study unrealistically quickly. We measure response speed using the average number seconds spent on each survey item (seconds per item, or SPI). We judge a response to be unrealistically fast if its SPI value is less than 1. SPI calculations will exclude optional items.
2. A respondent does not complete at least half of the items in the baseline survey.
3. A respondent provides off-topic, non-sensical (e.g., random or gibberish words), or non-English responses to an open-ended question in the baseline survey. The open-ended question follows a dictator game (that is not part of the pre-registered portion of the study) and asks respondents what they hoped to accomplish by acting as they did during the dictator game. The specific nature of this question will make it straightforward to detect off-topic responses. A comment must be judged as off-topic, non-sensical, or non-English by two members of the research team to be excluded.
4. A respondent does not agree to continue with the study when asked if they wish to continue taking part in the study at the end of the baseline study, or in private correspondence with the researchers.
5. A respondent completes the baseline study using an IP address from outside Canada or the United States, or that appears to originate from a VPS or other suspicious source.
6. Technical difficulties prevent a respondent from completing the baseline study.

Because obtain sufficient power to detect effects is a central aim of this study, the investigators will replace respondents who are removed for reasons 1-5 in a rolling fashion. In the case of technical failure (#6), the first approach will be to resolve the issue and administer the baseline survey to the same participant. If this is not possible, a replacement will be recruited.

Contacts/Locations

Central Contact Person: Andrew Miles, PhD
Telephone: 6474473212
Email: andrew.miles@utoronto.ca

Central Contact Backup: Christos Orfanidis, MA
Telephone: 6473850820

Email: c.orfanidis@mail.utoronto.ca

Study Officials: Andrew Miles, PhD
Study Principal Investigator
University of Toronto

Locations: **Canada, Ontario**
Online, Amazon's Mechanical Turk
Toronto, Ontario, Canada, M5S 2J4
Contact: Andrew Miles, PhD andrew.miles@utoronto.ca

IPDSharing

Plan to Share IPD: Yes
Using an OSF repository, other researchers and readers of the published study will have access to the IPD.

Supporting Information:
- Study Protocol
- Statistical Analysis Plan (SAP)
- Informed Consent Form (ICF)
- Analytic Code

Time Frame:
After the publication of the paper and perpetually. The investigators plan to publish the study as a registered report, so study information will also be available as part of the report protocol.

Access Criteria:
URL: https://osf.io/63yg9/

References

Citations: Morgan J, Farsides T. Measuring Meaning in Life. J Happiness Stud. 2009;10: 197-214. doi:10.1007/s10902-007-9075-0

Kohout FJ, Berkman LF, Evans DA, Cornoni-Huntley J. Two shorter forms of the CES-D (Center for Epidemiological Studies Depression) depression symptoms index. J Aging Health. 1993 May;5(2):179-93. PubMed 10125443

Bjelland I, Dahl AA, Haug TT, Neckelmann D. The validity of the Hospital Anxiety and Depression Scale. An updated literature review. J Psychosom Res. 2002 Feb;52(2):69-77. Review. PubMed 11832252

Lyubomirsky S, Lepper HS. A Measure of Subjective Happiness: Preliminary Reliability and Construct Validation. Soc Indic Res. 1999;46: 137-155.

Kennedy R, Clifford S, Burleigh T, Waggoner PD, Jewell R, Winter NJG. The shape of and solutions to the MTurk quality crisis. Polit Sci Res Methods. 2020; 1-16. doi:10.1017/psrm.2020.6

Wood D, Harms PD, Lowman GH, DeSimone JA. Response Speed and Response Consistency as Mutually Validating Indicators of Data Quality in Online Samples. Soc Psychol Personal Sci. 2017;8: 454-464. doi:10.1177/1948550617703168

Links:

Available IPD/Information:	Type: Individual Participant Data Set<br>URL: https://osf.io/63yg9/<br>Not yet available, but will be posted once study is complete.<br><br>Type: Study Protocol<br>Will be available when the study is published as a registered report.<br><br>Type: Statistical Analysis Plan<br>URL: https://osf.io/63yg9/<br>The investigators intend to publish this study as a registered report. The analysis plan is current as of this registration, but will likely change during the review process.<br><br>Type: Informed Consent Form<br>URL: https://osf.io/63yg9/<br><br>Type: Analytic Code<br>URL: https://osf.io/63yg9/<br>Will be posted after study is complete.
